# Perceptions of Obesity Among Healthcare Professionals and Policy Makers in 2023: Results of the Global OPEN Survey

**DOI:** 10.1002/osp4.70033

**Published:** 2025-01-08

**Authors:** John B. Dixon, Rohana Abdul Ghani, Paolo Sbraccia

**Affiliations:** ^1^ Iverson Health Innovation Research Institute Swinburne University of Technology Melbourne Australia; ^2^ Department of Internal Medicine Faculty of Medicine Universiti Teknologi MARA Sungai Buloh Malaysia; ^3^ Department of Systems Medicine University of Rome Tor Vergata Rome Italy

**Keywords:** healthcare policy, healthcare practitioner, obesity, obesity bias, survey

## Abstract

**Objective:**

Obesity is a disease with severe health impacts on individuals and economic impacts on society, yet healthcare practitioners (HCPs) and policy makers often fail to address it. This survey was conducted to examine current global obesity care and perceptions influencing care delivery among HCPs and healthcare decision makers (HC DMs).

**Methods:**

A survey with a cross‐sectional design was conducted among 1200 HCPs (primary care providers, endocrinologists, cardiologists, and nurses) and 414 HC DMs from eight countries across five continents. Respondents' perceptions of obesity, characteristics of patient populations, obesity management practices, and obesity‐related healthcare policies were collected. Surveys were administered online from June–July 2023. All respondent data were anonymized.

**Results:**

Among HCPs, 26.4% and 29.0% of HC DMs considered obesity a chronic disease, and 44.6% of HCPs reported that obesity was recorded as a chronic disease in patients' medical records. The pattern of responses was consistent across countries and professional roles. Obesity care approaches focused on lifestyle concerns. HCPs and HC DMs appeared to overestimate the provision of obesity‐related medical care for affected patients.

**Conclusion:**

These results corroborate prior findings that many HCPs do not consider obesity a disease, which hinders initiation of appropriate treatment, and also highlight challenges in obesity management, including gaps in obesity guidelines and accessibility to healthcare. These findings may help guide education and outreach by health authorities as well as HCPs.

## 1 Introduction

Nearly half (43%) of the global population has obesity or is overweight and at risk of obesity [1]. Persons living with obesity face major health consequences ranging from cardiovascular disease and diabetes to chronic kidney disease, cancer, and digestive disorders, along with associated risks of morbidity and mortality [2, 3]. In addition to posing serious health risks to individuals, obesity is associated with substantial direct and indirect costs, equivalent to nearly 2% of the global average gross domestic product [4]. To address these challenges, in 2023 the World Health Organization (WHO) developed a health service delivery framework for obesity that follows a chronic care approach and integrates obesity prevention and management into existing service delivery frameworks to improve obesity care globally [5].

Obesity was first classified as a disease by the World Health Organization in 1948 and more recently has been designated as a chronic disease by multiple medical societies [6, 7, 8, 9, 10]. To clarify the clinical and biological criteria for the diagnosis of obesity, the Lancet Commission on Obesity recently announced an effort to reach a consensus on the definition of obesity based on increased adipose tissue and the onset of complications (cardiorenal, metabolic, respiratory, musculoskeletal, and inflammatory disorders, along with increased mortality risk) as major characterizing features, with a natural history that involves progressive pathologic alterations in neuroendocrine signaling pathways [9]. This effort follows many innovative advances to move beyond BMI and anthropometrics measures to include the risks, complications, and functional impairment of obesity in the diagnosis and staging of this chronic condition [6, 11, 12, 13].

Despite the severe impact of obesity on individuals and society, healthcare practitioners (HCPs) frequently fail to address obesity with their patients [14, 15, 16, 17, 18]. Moreover, shame and internalized stigma often prevent people with obesity (PWO) from seeking medical care for even general health concerns [19]. The belief that obesity is a personal failing rather than a disease with pathophysiologic origins may contribute to these gaps. In addition, a lack of consensus on the definition of obesity—whether it is itself a disease or is “merely” a disease risk factor—may deter HCPs from addressing obesity management with their patients [9]. Lack of training on obesity management and poor insurance reimbursement rates also contribute to the lack of obesity management by HCPs [20, 21].

The global Obesity Policy Engagement Network (OPEN) was established in 2018 as a partnership program between the Obesity Society, the European Association for the Study of Obesity, the World Obesity Federation, the European Coalition for People Living with Obesity, the Global Obesity Patient Alliance, and Novo Nordisk with the goal of improving obesity care internationally. To better understand how current obesity care is provided worldwide and investigate perceptions that influence the delivery of care, OPEN conducted a survey of HCPs and healthcare decision makers (HC DMs) from eight nations spanning five continents and representing a variety of socioeconomic and cultural settings. The survey was conducted between 3 June 2023 and 18 July 2023.

## 2 Methods

### 2.1 Study Design

A cross‐sectional survey design was employed to collect data from a diverse sample of HCPs and HC DMs in each nation surveyed. The surveys used were de novo instruments (Supplementary Materials, Survey Instruments) developed by the OPEN Secretariat and CensusWide (London, UK), an international market research consultancy that conducts qualitative and quantitative research in accordance with the European Society for Opinion and Marketing Research (ESOMAR) principles.

### 2.2 Survey Development and Data Collection Methods

An outline of survey topics and proposed questions, which were developed based on themes explored in the Action IO survey [14], were reviewed by HCPs and obesity experts from Canada, Brazil, Italy, Germany, Spain, Singapore, Israel, and the United States. To ensure relevance and validity of topics and questions, additional verbal and written input was collected from HCPs and obesity experts from Italy, Germany, Canada, Brazil, and European level organizations, and then collated and incorporated into final questionnaires. The questionnaires were assessed and further refined by CensusWide to ensure they were easy to understand and delivered complete and robust results.

The final HCP questionnaire consisted of 24 questions that collected information on respondents' demographic characteristics, type of practice/specialty, characteristics of their patient population, perceptions of obesity, and obesity management practices. The HC DM questionnaire consisted of 22 questions and likewise collected demographic information and respondents' perceptions of obesity and their obesity‐related healthcare policies and practices. Surveys were administered in the native languages of the countries surveyed (i.e., English, German, Italian, Malay, Portuguese, Spanish, and Turkish).

### 2.3 Survey Population

The online survey utilized single stage sampling of individuals. The sample size of 1200 HCPs was determined based on an expected confidence interval of 2.82 at a 95% confidence level, whereas the sample size of 400 HC DMs was determined based on an expected confidence interval of 4.81 at a 95% confidence level.

The HCP survey population included general practitioners (GPs) and primary care providers (PCPs), cardiologists, endocrinologists, and practice nurses from Australia, Brazil, Canada, Germany, Italy, Malaysia, Spain, and Turkey. Surveyed HC DMs from the same nations included commissioners, heads of department, hospital, clinic, or practice, and individuals who sit on national/regional health committees.

Respondents were recruited from pre‐existing panels whose members were recruited via healthcare forums and events and by word of mouth. Upon joining, panelists answered a profile questionnaire to enable targeted recruitment into surveys. For the present survey, HCPs were included if ≥ 10% of their patient population was living with obesity and they were a GP/PCP, cardiologist, or endocrinologist aged ≥ 30 years or a practice nurse aged ≥ 22 years. At least 25 respondents from each specialty in each country were recruited for the survey. HC DMs were included if they confirmed working in the healthcare field, reported an integral role in the decision process for implementation of obesity care, and held a leadership position within their institution or policy‐making group. Demographic information on sex/gender and race/ethnicity was not collected. Other than the HCP specialty quota (*n* = 25 per specialty per country), no specific recruitment quotas were set.

### 2.4 Survey Administration

Surveys were administered online via a website interface from 3 June 2023 to 18 July 2023. Survey respondents were recruited via a double opt‐in validation procedure. First, potential respondents were invited via email to participate based on information from the profiling questionnaire they filled out when they joined the panels. Next, potential respondents passed through a non‐leading screening process to ensure they met inclusion criteria prior to being allowed to answer the surveys. Regular data cleaning was conducted throughout the survey period to ensure genuine responses.

Respondents received an incentive to respond to the survey, which consisted of points that could be redeemed as purchase vouchers or charity donations. Incentives were collected upon successful completion of the questionnaire and after passing security checks (such as IP address checks). Successful completion was defined as a completed survey that met quality assurance standards, including answering survey questions within the expected estimated time of completion (not more quickly) in a pattern consistent with genuine answers (e.g., not clicking on only the top or bottom answer throughout).

### 2.5 Ethical Considerations

All responses were treated anonymously, in strict confidentiality and in line with the 2018 General Data Protection Regulation (GDPR). No personally identifiable information was linked to the results. All respondents were anonymous from the point of entering the survey.

### 2.6 Statistical Analysis Methods

Descriptive and inferential statistics were used to analyze the data which were compiled in a spreadsheet. Survey findings are reported descriptively as percentages.

In the case of questions where the answer options were ranges (e.g., 1%–10%, 11%–20%, etc.), overall mean values were determined by assigning the value of each range as its midpoint (e.g., 5.5%, 15.5%) and then determining the overall mean based on the proportions of respondents selecting each range.

## 3 Results

### 3.1 Survey Respondents: Demographic Information, Training, and Practice Make‐Up

A total of 1200 HCPs from Australia, Canada, Brazil, Spain, Italy, Malaysia, Turkey and Germany (*n* = 150/country) and 414 HC DM respondents (∼50 per country) participated in the survey. Demographic characteristics are provided in Table S1.

**FIGURE 1:**
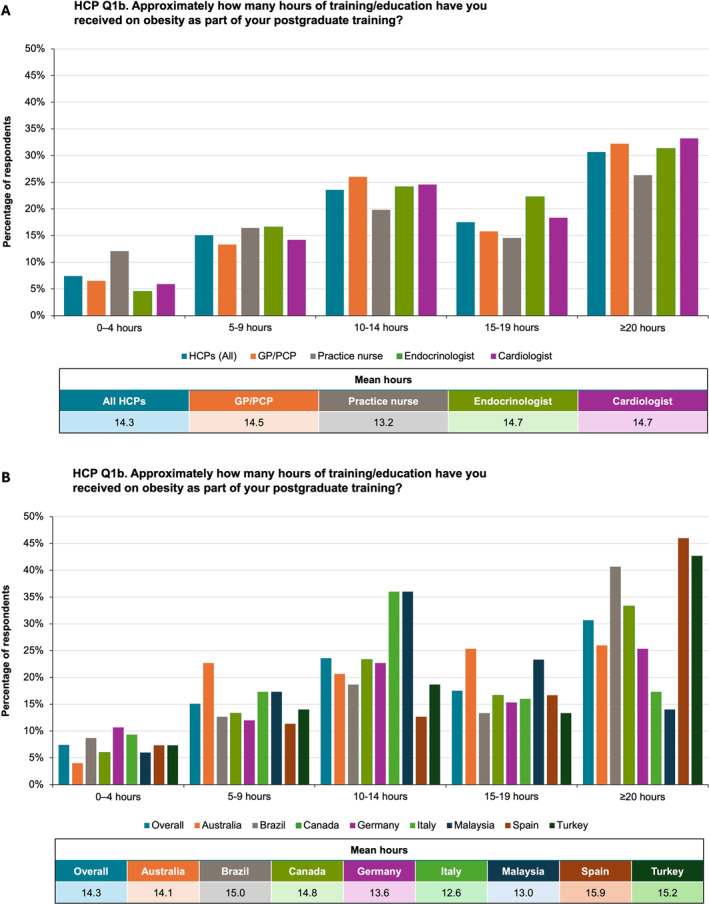
Proportions of healthcare practitioners (HCPs) reporting 0–4, 5–9, 10–14, 15–19, and ≥ 20 h of postgraduate education on obesity, with mean numbers of hours, by specialty (A) and country (B). GP, general practitioner; PCP, primary care provider.

Across specialties and countries, the mean number of hours of HCP postgraduate education on obesity ranged from 13 to 16 h; approximately a third of the HCPs reported receiving ≥ 20 h. Practice nurses consistently reported receiving fewer hours of obesity training than physicians (Figure 1, Table S1). HCPs from Brazil, Spain, and Turkey were more likely than HCPs from other nations to report receiving ≥ 20 h (Figure 1, Table S1).

Respondents reported that substantial proportions of their patients living with obesity had concomitant medical conditions, including cancer, diabetes, cardiovascular disease, gastrointestinal disorders, physical complications (mobility limitations or complications), malnutrition (micronutrient deficiencies, sarcopenic obesity, etc.), skin conditions, and psychological conditions (disordered eating, attention deficit hyperactivity disorder, etc.) (Figure S1).

### 3.2 Perceptions, Beliefs, and Attitudes About Obesity and People With Obesity

**FIGURE 2:**
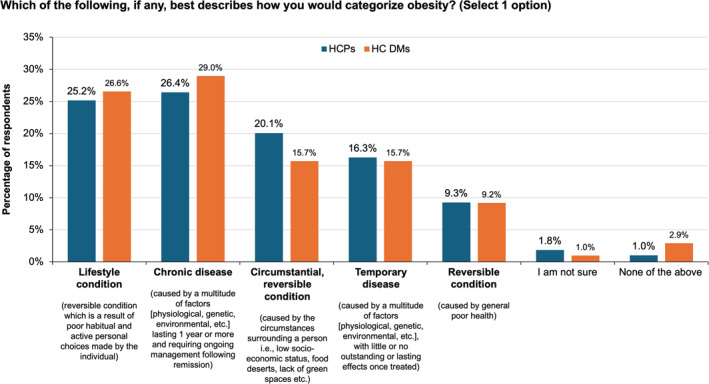
Categorization of obesity by healthcare practitioners (HCPs) and healthcare decision makers (HC DMs).

Only about a quarter of HCPs reported categorizing obesity as a chronic disease (26.4%), where *chronic disease* was defined as one that is caused by a multitude of physiological, genetic, and environmental factors lasting ≥ 1 year and requiring ongoing management following remission. Another quarter perceived it as a lifestyle condition (25.2%), which was defined as a reversible condition that is a result of poor habitual and active personal choices made by the individual. Categorization of obesity by HC DMs was similar (Figure 2).

**FIGURE 3:**
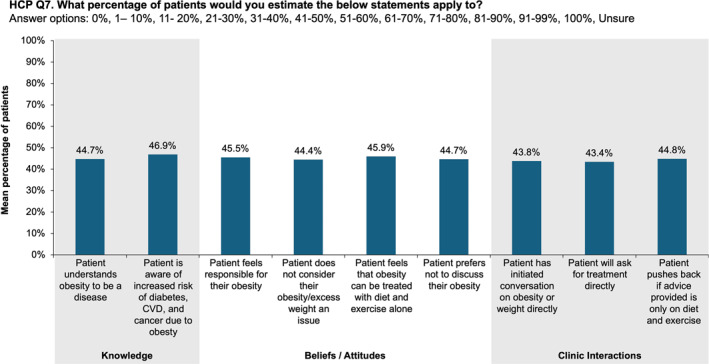
Healthcare practitioner (HCP) estimates of their patients' knowledge, beliefs, and attitudes about obesity and about obesity‐related discussions in the clinic/practice. Mean percentages were determined based on the proportions of respondents selecting each numerical answer option (i.e., 0%, 1%–10%, etc.) and using the midpoint of each range as the value for options that were ranges. Less than 2% of respondents answered “unsure” for each category. CVD, cardiovascular disease.

As shown in Figure 3, HCP respondents reported that less than half of their patients understood obesity to be a disease or were aware that their risk of diabetes, cardiovascular disease, or cancer was increased by obesity. With regard to patient beliefs and attitudes, HCPs estimated that less than half of their patients felt responsible for having obesity and a similar proportion preferred not to discuss obesity. HCPs projected that slightly more than half of their patients thought that obesity is not considered an issue and could be treated with diet and exercise alone. However, HCPs also reported that almost half of their patients with obesity initiated conversations on obesity or weight and directly requested obesity treatment. In addition, HCPs estimated that nearly half of patients would “push back” if the HCP provided only advice on diet and exercise (Figure 3).

Two thirds of HCPs agreed there is a greater need for health literacy on obesity in the following settings: in the general public to inform decisions and actions toward people living with obesity (66.4%); in people at risk of or living with obesity to inform decisions and actions regarding prevention, treatment, and management of obesity (66.3%); and in the medical community to inform decisions and actions regarding prevention, treatment, and management of people at risk or living with obesity (65.8%).

When asked about their own attitudes toward obesity and PWO, 41.9% of HCPs agreed that “obesity is a result of personal and conscious decisions to perform a behavior that increases the risk of obesity” and 36.8% agreed that “people are responsible for managing obesity on their own”; 23.2% and 24.5% of HCPs disagreed with these statements, respectively (Figure S2). Findings were generally consistent across specialties and countries, although Malaysia was the only nation in which more than half of HCPs agreed that obesity was the result of personal decisions and that patients should manage it on their own (54.7% and 56.0%, respectively) (Figure S2).

Obesity bias was commonly reported in the survey; 39.8% of HCPs reported they felt bias against PWO, and 44.8% reported bias against PWO by professional colleagues. Although 64.3% of HCPs agreed that PWO deserve the same respect, care, and treatment as other people with chronic diseases, a non‐negligible proportion of 9.2% disagreed with this statement.

### 3.3 Use of Obesity Clinical Practice Guidelines

Overall, 47.3% of HCPs reported consulting obesity clinical practice guidelines (CPGs), with the highest rate among endocrinologists (55.1%). However, 28.2% of HCPs reported that the available obesity CPGs were “inadequate” and 13% reported not consulting them at all (Figure S3A). A majority of HCPs from Australia (53.3%), Brazil (63.3%), and Malaysia (52.7%) reported that they consult obesity CPGs, while < 40% of HCPs from Canada and Turkey reported doing so (Figure S3B). In contrast, a majority of HC DMs from Canada (56.0%) and Australia (53.5%) reported that dedicated obesity CPGs were available and regularly consulted in their country, but most HC DMs from other nations reported CPG‐related shortcomings, including that the CPGs themselves were inadequate or there were insufficient resources to implement CPG recommendations (Figure S3C). Most HC DMs reported that their country's obesity‐related CPG covered nutrition (55.6%), treatment (48.3%) and diagnosis (48.1%). However, HC DMs reported low figures on disease progression (34.3%), pharmacotherapy (25.6%), and surgical intervention (31.9%) (Figure S3D).

### 3.4 Diagnosis and Management of Obesity

**FIGURE 4:**
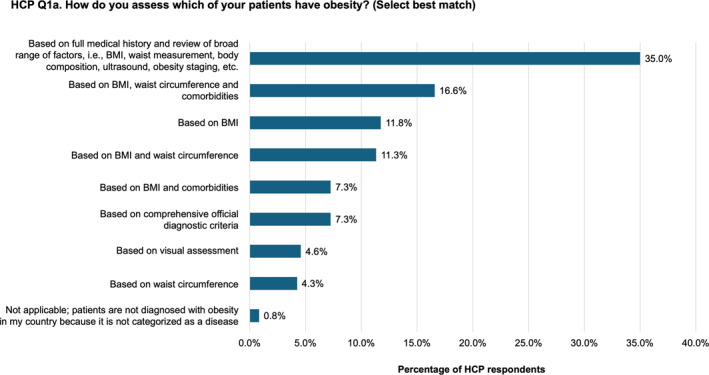
HCP‐reported methods of assessing whether patients have obesity. BMI, body mass index; HCP, healthcare practitioners.

Approximately a third of HCPs reported that they assess for obesity based on a full medical history and review of a broad range of factors, including body mass index (BMI), waist circumference, body composition, ultrasound, and obesity staging, etc (Figure 4). Assessment patterns among the different specialties and countries were broadly similar (Figure S4).

Overall, HCPs reported discomfort discussing obesity and related topics with their patients (‘What percentage of your patients do you feel comfortable and actively discuss obesity with?’; Figure S5A). HCPs were most willing to discuss obesity in a general manner, reporting that they “feel comfortable discussing obesity” with a mean of 48.8% of their patients. They were more willing to actively discuss obesity with patients who were at risk, based on indicators such as BMI cut‐offs (48.1% of patients), obesity indicators (48.0%), and comorbidities (48.6%), rather than on visual assessment (44.7%). Among different specialties, endocrinologists were more comfortable or more likely to actively engage in discussions about obesity than their counterparts (Figure S5A). Relative to HCPs from other nations, Brazilian HCPs were more likely to report being comfortable or actively addressing obesity with their patients (Figure S5B).

HCPs reported an overall mean of 44.6% of patients with a diagnosis of obesity had it listed in the patient's medical record as a *chronic disease*, with mean estimates ranging from 39.8% in Turkey to 54.0% in Spain (Table S2). Mean values among the different specialties were similar to the overall mean (Table S2).

**FIGURE 5:**
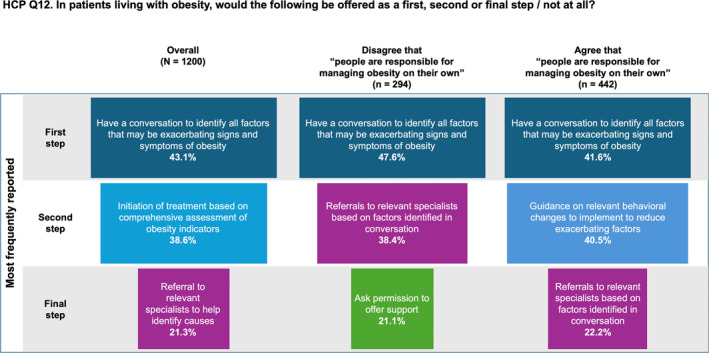
Tornado diagrams showing the most frequently selected answers to what healthcare practitioners (HCPs) would offer as the first, second, and final obesity management step in the overall survey population and in subgroups who disagreed and agreed, respectively, that “people are responsible for managing obesity on their own.” See Question 12 of the HCP Survey (Supplementary Materials) for the full list of possible answers.

As shown in Figure 5, the most frequently chosen first step in the management of obesity within the overall population of HCPs was to “have a conversation with patients to identify all factors that may be exacerbating signs and symptoms of their obesity (i.e., mental health, stress, poor sleep, medication, etc.),” (43.1%), followed by initiation of treatment based on comprehensive assessment of obesity indicators (38.6%), and referral to specialists (21.3%). Data were divided into two groups of HCPs: those who agreed with the statement “people are responsible for managing obesity on their own” and those who disagreed with this statement. Both groups seemed to agree with starting a conversation as the first step in obesity management, albeit a smaller number in the former (41.6% vs. 47.6%, respectively). However, the second step differed considerably, with the group who agreed that patients were responsible for managing obesity choosing guidance on behavioral changes as the favored second step (40.5%), followed by referral to specialists as the third step (22.2%). The HCPs who disagreed with the statement chose specialist referral as the preferred second management step (38.4%) and permission to offer support as the third step (21.1%). HCPs reported referring less than half of PWO to specialist care, including nutritionists (49.0%), endocrinologists (44.7%), cardiologists (45.4%), mental health counseling (43.4%), or treatment for eating disorders (45.4%), among other services (Figure S6).

**FIGURE 6:**
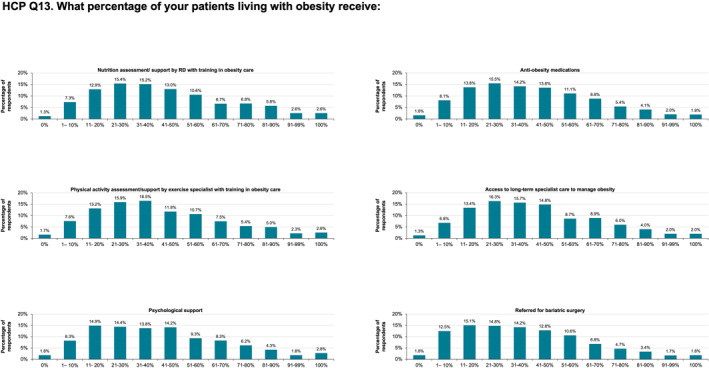
Percentage of healthcare practitioners (HCP) reporting the percentage of their patients with obesity who receive various obesity interventions. *Specialist care* was defined as “endocrinologist, cardiologist, obesity nurse specialist, etc.” RD, registered dietitian, or professional nutritionist.

HCPs reported recommending consultations for lifestyle interventions to less than half of their patients (Figure 6). Responses were distributed across the full range of options (from 0% to 100% in 10% increments) but tended to be skewed so that less than half of patients received advice on nutritional advice, physical activity support, and anti‐obesity medications (Figure 6). Response trends indicated that psychological support was offered to fewer patients than other interventions. Approximately 16% of HCPs reported prescribing anti‐obesity medications and making referrals to specialist care to 21%–30% of their patients. The mean percentage of patients offered any one of 10 nonsurgical interventions ranged between 41% and 43%, and HCPs reported that an average of ∼37% of patients with obesity had been referred for or had undergone bariatric surgery (Figure S7A). Compared with the other specialties surveyed, cardiologists offered obesity interventions to more patients, followed by endocrinologists (Figure S7A), and interventions seemed to be more commonly offered in Brazil and Australia than in other nations (Figure S7B).

### 3.5 Obesity‐Related Public Policy

**FIGURE 7:**
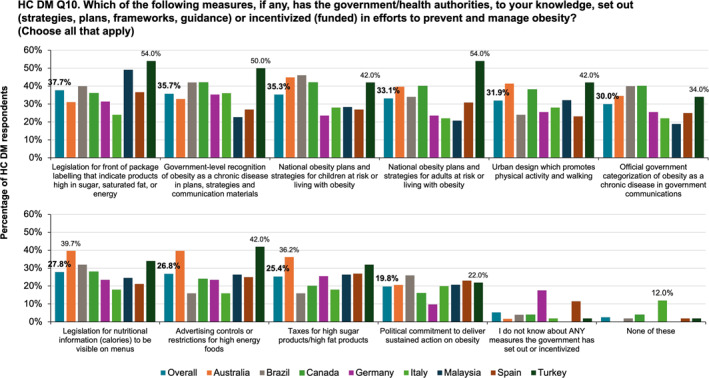
Healthcare decision‐maker (HC DM) reporting on government measures to prevent and manage obesity.

As shown in Figure 7, overall, 35.7% of HC DMs reported government‐level recognition of obesity as a chronic disease, and 30.0% reported official government categorization of obesity as a chronic disease. National obesity plans for children and adults and urban design plans promoting physical activity were reported by a third of HC DMs. However, only one in 5 HC DMs reported political commitment to sustained action on obesity. Turkish HC DMs were more likely than their counterparts from other nations to report government support for the management of obesity.

**FIGURE 8:**
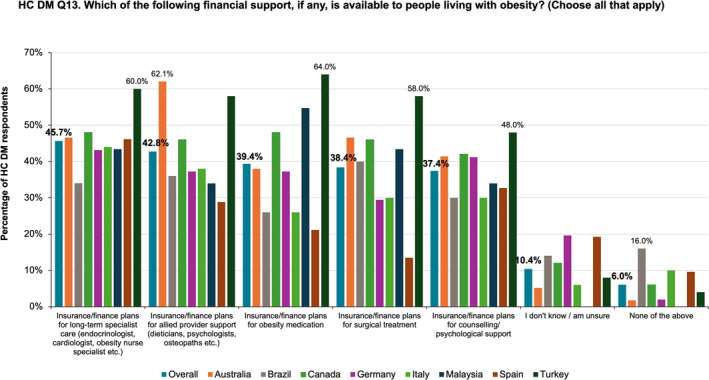
Healthcare decision‐maker (HC DM) reporting on insurance coverage or other financial support for obesity specialist care or interventions.

Financial support in terms of insurance coverage or finance plans for long‐term specialists (e.g., endocrinologist, cardiologist, obesity specialty nurse) was reported by 45.7% of HC DMs overall, and anti‐obesity medication coverage was reported by 39.4% of HC DMs (Figure 8). Financial planning for the management of obesity was significantly discrepant between the surveyed nations, with the highest proportion of HC DMs from Turkey reporting favorable support. Australian HC DMs were more likely than their counterparts to report financial support for allied providers such as dietitians, psychologists, and osteopaths.

## 4 Discussion

The findings of the surveys developed and performed by the OPEN Secretariat and CensusWide have added to the understanding of barriers to the effective management of obesity. The pattern of responses was consistent across all eight countries and HCP roles surveyed, and responses were also consistent between HCPs and HC DMs, when they were asked the same questions.

HCPs reported that most of their patients with obesity live with complications, such as cancer, diabetes, cardiovascular disease, gastrointestinal disorders, etc., consistent with observed strong associations between obesity and a wide range of metabolic, cardiorenal, and other diseases, including cancer [22, 23, 24, 25]. A recent epidemiologic study showed that compared to people without obesity (using BMI values of 18.9–24.9 kg/m^2^), PWO are up to 5 times more likely to have a second disease, 9 times more likely to have a third disease, and 13 times more likely to have a fourth disease [25]. However, in the present survey, less than 30% of HCPs and HC DMs surveyed considered obesity a chronic disease, and less than half reported that obesity was recorded as a chronic disease in patients' medical records. Small numbers of respondents also reported using relevant basic metrics such as BMI or waist circumference. Meanwhile, HC DMs and many HCPs reported believing that the necessary infrastructure for managing obesity is already in place, but management options are often not available to or accessible by affected people. The majority of participants in the present survey considered obesity a reversible condition that could be managed by addressing personal behaviors and an individual's surrounding circumstances, findings that show little change in attitude from previous HCP surveys [14, 15, 16]. This may be related to limited and inadequate training, with behavioral, dietary, and physical activity discussions dominating, while the obesity pathophysiology, regulation of energy balance, and stigma are poorly addressed if at all [26, 27, 28].

HCPs in the present survey reported an average of 41% and 37% of their patients were using obesity medications or had undergone metabolic/bariatric surgery, respectively. These findings seem discordant with real world conditions within the countries surveyed. The few available reports indicate an uptake of obesity medications by less than 2% of eligible populations [29, 30, 31]. A global survey estimating the annual uptake of metabolic/bariatric surgery among patients with obesity and type 2 diabetes included six of the countries in the current survey; the estimated annual uptake of surgical interventions varied from a high of 1.3% in Australia to 0.05% in Turkey [32].

Recent findings have revealed that treatment of clinical obesity extends beyond eating less and moving more [33]. The over‐estimation of the efficacy of therapeutic interventions solely based on patients' education and lifestyle modification is responsible for extreme therapeutic inertia within many current obesity guidelines and throughout health services. The present survey confirmed the inadequacy of many obesity management guidelines, with only 26% of respondents reporting their obesity‐related CPGs cover obesity pharmacotherapy, 34% reporting CPG coverage of disease progression, and 32% reporting coverage of surgery in their CPG. These gaps generate and perpetuate weight stigma [33, 34].

The present findings reflect widespread myths and perceptions of obesity, which continue to dominate the narratives of “eating less and moving more.” Evidence has shown that focusing on nutrition and physical activity alone promotes obesity stigma, generates internalized stigma, and exacerbates the biopsychosocial complications of obesity [35, 36, 37, 38, 39]. A recent survey of adults enrolled in behavioral weight management programs showed that weight stigma in healthcare is prevalent and experienced similarly across six Western countries. Furthermore, this internalized weight bias has negative implications for healthcare [40]. The findings presented here are consistent with previous surveys showing that HCPs have a poor understanding of obesity with its biopsychosocial complications and do not generally address obesity as a disease per se [14, 15, 16, 17, 18, 41, 42, 43]. The current survey findings also suggest a high level of weight bias, with 40% of HCPs and 29% of HC DMs indicating they hold biases toward PWO, and 45% and 39%, respectively, agreeing that their colleagues hold such biases.

The Action IO study of over 14,000 PWO and 2700 HCPs from 11 countries (Australia, Chile, Israel, Italy, Japan, Mexico, Saudi Arabia, South Korea, Spain, UAE, and UK), along with additional ACTION studies conducted in the United States and Canada, found that the majority of HCPs and PWO believed it was a patient's responsibility to manage obesity, mainly with dietary interventions and lifestyle changes [14, 17, 18]. These surveys were designed to compare attitudes and perceptions of PWO and HCPs and demonstrate communication misalignments and gaps, rather than focus strictly on the perceptions and attitudes of HCPs and HC DMs regarding obesity, as in the present study. Notably, 67% of ACTION IO HCPs were regarded as specialists in weight management, whereas the HCPs in the present survey may be considered as generalists, because the only inclusion requirement was that ≥ 10% of patients in their practice be PWO. Notably, less than half of the HCPs in the present survey provided their patients with nutrition, physical activity, and mental health assessment or support from trained professionals in obesity care (i.e., registered dietitians, exercise specialists, psychologists, etc).

Action IO found a large discrepancy between HCPs and PWO in their perceptions of the consultation expectation and outcomes [14]. The current survey confirms the views of the HCPs in the Action IO study, by demonstrating potential barriers within the consultation and the risk that the interaction may impair engagement and exacerbate damaging internalized weight stigma. Endocrinologists participating in the present survey were more likely to initiate a conversation regarding obesity, but cardiologists were more likely to initiate treatment. Perhaps this finding reflects therapeutic inertia in which treatment was only initiated after the onset of obesity‐related cardiovascular complications.

Overall, the responses of HCPs and HC DMs in the present survey suggest a sense of complacency and futility. Changing the narrative will be complex as the beliefs and perceptions regarding obesity are widely held throughout society. The scientific, biological, and clinical evidence regarding obesity challenges a common, deeply held “self‐evident” narrative not only among PWO but also among HCPs and HC DMs. Addressing the misleading issues surrounding obesity is critical to changing the narrative, improving health outcomes, and improving public health messaging [40, 44]. As with any form of misperception, the path toward inclusion starts with widespread awareness and empathy [44]. HCPs and HC DMs can play a major role in increasing the awareness and health literacy regarding obesity among HCPs and the public, respectively. HC DMs are also well placed to prioritize obesity and craft and implement holistic obesity action plans that are consistent with the WHO obesity health service delivery framework. These efforts will eventually reduce the prevalence of overweight and obesity as well as reduce obesity‐related complications and improve the overall health of PWO.

Several factors may limit interpretation of these survey findings. First, the results may not be generalizable to all HCPs or HC DMs because the respondents to this survey were recruited from pre‐existing panels and met criteria for experience with obesity (at least > 10% of their patient population having obesity or played an integral, decision‐making role in the implementation of obesity care). Second, the wording of the questionnaire may have generated a difference in understanding between respondents in different countries and health services. There may have also been response bias with misinterpretation of questions and/or response fatigue as several questions in the HCP and HC DM surveys contained multiple sub‐questions. Lastly, the sample size of HCPs and HC DMs from individual countries and specialties was low, which may not have been representative of these populations' overall beliefs or practices; however, the consistency of responses across nations and specialties supports these conclusions.

## 5 Conclusion

This survey highlighted the reluctance among HCPs to consider obesity as a disease, which subsequently hinders the initiation of appropriate treatment and further perpetuates obesity stigma. It also demonstrated challenges in obesity management, including the lack of practicality in available guidelines and limited accessibility to evidence‐based management/care. These findings would be useful for relevant authorities, including patient‐led advocacy groups, HCP associations, HC DM administrators, researchers, and government officials, to better understand the gaps and deficiencies in the delivery of effective obesity care in their respective countries.

## Conflicts of Interest

J.B.D. reports consulting fees from Reshape Lifescience and Nestle Health Science; payment or honoraria for lectures, presentations, speakers bureaus, manuscript writing or educational events from Novo Nordisk, Lilly, INova, Eurodrug Laboratories, HealthED, and Nestle Health Science Australia; support for attending meetings and/or travel from Novo Nordisk, Lilly, INova, Eurodrug Laboratories, and HealthED; participation on an Advisory Board for Reshape Lifescience, Nestle Health Science, Lilly, and Novo Nordisk; and service as vice president of NACOS, a leader of the Obesity Collective, and a member of WIN. R.A.G reports service as editor‐in‐chief of the Journal of Clinical and Health Sciences, Universiti Teknologi MARA (UiTM). P.B. reports receiving consulting fees from Eli Lilly, Novo Nordisk, Pfizer, Boehringer Ingelheim, Bruno Farmaceutici, and Amryt (Chiesi); payment or honoraria for lectures, presentations, speakers bureaus, manuscript writing or educational events from Eli Lilly, Novo Nordisk, Bruno Farmaceutici, and Amryt (Chiesi); support for attending meetings and/or travel from Eli Lilly, Novo Nordisk, and Bruno Farmaceutici; and participation on an Advisory Board for Eli Lilly, Novo Nordisk, Pfizer, Boehringer Ingelheim, Bruno Farmaceutici, and Amryt (Chiesi).

## Supporting information

Supporting Information S1
