# Supplementary material for: Perceptions of Obesity Among Healthcare Professionals and Policy Makers in 2023: Results of the Global OPEN Survey
Source: Obes Sci Pract. 2025 Jan 8;11(1):e70033. doi: 10.1002/osp4.70033 (PMC11707619; doi:10.1002/osp4.70033)

SUPPLEMENTAL MATERIALS FOR

Perceptions of Obesity Among Healthcare Professionals and Policy Makers in 2023: Results of the Global OPEN Survey

John B. Dixon, Rohana Abdul Ghani, Paolo Sbraccia

Table of Contents

[OPEN Models of Care Survey—HCPs 3](#_Toc178178205)

[OPEN Models of Care Survey—Decision makers 22](#_Toc178178206)

[Table S1. Demographic characteristics of survey population. 38](#_Toc178178207)

[Figure S1. Healthcare practitioner (HCP) responses to: “Qii. What proportion of your patients living with obesity have comorbidities for which they receive treatment and/or specialist care?” 40](#_Toc178178208)

[Figure S2. Healthcare practitioner (HCP) responses to: “Q15. To what extent do you agree or disagree with the following statements?” with regard to statements about HCP attitudes about obesity. 41](#_Toc178178209)

[Figure S3. Use of clinical practice guidelines (CPG)s. 42](#_Toc178178210)

[Figure S4. Healthcare practitioner (HCP)–reported methods of assessing whether patients have obesity by specialty (A) and country (B). 44](#_Toc178178211)

[Table S2. Mean percentages of HCPs reporting that patients’ diagnoses of obesity were or were not documented in their chart as a “chronic disease.”^a^ 45](#_Toc178178212)

[Figure S5. Mean percentages of patients with whom healthcare practitioners (HCPs) report feeling comfortable discussing obesity or actively engage in discussions about obesity. 46](#_Toc178178213)

[Figure S6. Mean percentages of patients with obesity receiving referrals for the services listed as reported by healthcare practitioners (HCPs). 48](#_Toc178178214)

[Figure S7. Mean percentages of patients with obesity receiving the obesity interventions listed as reported by healthcare practitioners (HCPs). 49](#_Toc178178215)

# OPEN Models of Care Survey—HCPs

Qa. How old are you? TO BE REPORTED AS:

- 22-30
- 31-40
- 41-50
- 51+

**END IF AGED UNDER 22**

Qb. Which best describes your job title?

- Endocrinologist
- General Practitioner GP/ Primary care provider (PCP)
- Cardiologist
- Practice nurse
- None of the above [END]

**QUOTAS**

MINIMUM N=25 PER JOB TITLE

[END] IF RESPONDENT TICKS ANY OTHER JOB TITLE BUT ‘Practice Nurse’ AND AGED UNDER 30

Qc. Thinking of the total number of your patients, how many of these have obesity?

- Less than 10% [END]
- 10-25%
- 26-50%
- 51-75%
- 76-99%
- 100%
- Not sure [END]

Caveat Q1a: If you answer an open response, please do it in English language.

**BOLD/UNDERLINE 'Engllish language'***

Q1a. How do you assess which of your patients have obesity? Select best match

- Based on visual assessment
- Based on BMI
- Based on weight circumference
- Based on BMI and weight circumference
- Based on BMI and comorbidities
- Based on BMI, weight circumference and comorbidities
- Based on full medical history and review of broad range of factors i.e. BMI, waist measurement, body composition, ultrasound, obesity staging etc.
- Based on comprehensive official diagnostic criteria
- Other, please specify
- Not applicable, Patients are not diagnosed with obesity in my country because there are no diagnostic criteria
- Not applicable, Patients are not diagnosed with obesity in my country because it is not categorised as a disease
- Not applicable, I do not diagnose people with obesity

Q1b. Approximately how many hours of training/education have you received on obesity as part of your medical education, post graduate training? *matrix*

Rows:

- Medical education
- Post graduate training Columns:
- Up to 4 hours
- 5-9 hours
- 10-14 hours
- 15-19 hours
- 20 hours or more
- Unsure/cannot remember

Q2. Which of the following, if any, best describes how you would categorise obesity? [Select 1 option]

- A lifestyle condition (reversable condition which is a result of poor habitual and active personal choices made by the individual)
- A circumstantial condition (reversable condition caused by the circumstances surrounding a person i.e., low socio-economic status, food deserts, lack of green spaces etc.)
- A condition (a reversable condition caused by general poor health)
- A disease (a temporary disease caused by a multitude of factors (physiological, genetic, environmental etc.), with little or no outstanding or lasting effects once treated
- A chronic disease (caused by a multitude of factors (physiological, genetic, environmental etc.) lasting 1 year or more and requiring ongoing management following remission
- I am not sure
- None of the above

Caveat Q3: If you answer an open response, please do it in English language.

**BOLD/UNDERLINE 'Engllish language'***

Q3. Are you aware of dedicated/specific obesity clinical practice guidelines (CPGs) (as opposed to guidelines on obesity included within another condition, i.e., diabetes) published within your health system and/or country? (Select 1 option)

- Yes, I am familiar with and have consulted our obesity CPGs
- Yes, I am aware of obesity CPGs but find them inadequate
- Yes, I am aware of obesity CPGs, but I have not consulted them
- Yes, please specify
- No, we do not have dedicated obesity CPGs
- Not applicable, I follow guidelines from a different disease area that has obesity guidance
- Not applicable, I follow obesity guidelines set out by my place of work
- I am unaware if we have obesity CPGs

Q4, What percentage of your patients would you estimate have received an official diagnosis of obesity and have it documented in their patient records? *MATRIX*

Rows:

- Patients have been diagnosed with obesity, but it is not documented as a chronic disease in their patient records (this also applies to patients where weight alone/BMI has been

documented in their records as part of routine checks)

- Patients have been diagnosed and obesity is documented in their patient records as a chronic disease

***SLIDING SCALE combination of % cannot exceed MUST ADD UP TO 100%***

Q5. What percentage of your patients do you feel comfortable and actively discuss obesity with?

*MATRIX*

Rows:

- Feel comfortable discussing obesity
- Actively discuss the risk of obesity with patients with relevant BMI cut-offs
- Actively discuss obesity with patients based on visual assessment
- Actively discuss obesity with patients at risk of obesity based on obesity indicators
- Actively discuss obesity with patients if the patient has or is at risk of other comorbidities

Columns:

- 0%
- 1 – 10%
- 11- 20%
- 21-30%
- 31-40%
- 41-50%
- 51-60%
- 61-70%
- 71-80%
- 81-90%
- 91-99%
- 100%
- Unsure

Q6. Thinking about financial incentives, how many of the following types of people are you incentivized to care for? *MATRIX*

Rows:

- People of non-Caucasian ethnicities diagnosed with obesity based on ethnicity-specific BMI cutoffs under 30 kg/m2 without comorbidities
- People of non-Caucasian ethnicities diagnosed with obesity based on ethnicity-specific BMI cutoffs under 30 kg/m2 with comorbidities
- People with BMI between 30 -34 kg/m2 without comorbidities
- People with BMI between 30-34 kg/m2 with comorbidities.
- People with BMI over 35 kg/m2 without comorbidities
- People with BMI over 35 kg/m2 with comorbidities
- People identified as being at risk of obesity based on comprehensive screening of obesity indicators (not specific to BMI)
- People diagnosed with obesity based on comprehensive diagnostic criteria (not specific to BMI)
- People living with obesity when deemed a risk factor for other conditions
- Other

Columns:

- 0%
- 1– 10%
- 11- 20%
- 21-30%
- 41-50%
- 51-60%
- 61-70%
- 71-80%
- 81-90%
- 91-99%
- 100%
- Unsure

Q7. What percentage of patients would you estimate the below statements apply to? *MATRIX* Rows:

- Patients with obesity will ask for treatment directly
- Patients understand obesity to be a disease
- Patients push back if advice provided is only on diet and exercise
- Patients feel that obesity can be treated with diet and exercise alone
- Patients feel they are responsible for their obesity
- Patients are aware of having an increased risk of diabetes, cancer, and cardiovascular disease due to their obesity
- Patients do not consider their obesity / excess weight an issue
- Patients prefer not to discuss their obesity
- Patients have been screened for obesity prior to the presence of visible signs and symptoms i.e., excess weight, reviewing a number of indicators of obesity
- Patients have received an early diagnosis (meaning detecting an individual who is living with obesity as early as possible based on signs)
- Patients have initiated conversation on obesity or weight directly
- Patients receive ongoing follow-up appointments to discuss their obesity
- Women living with obesity who hold excess weight and are pregnant receive maternal health counselling specifically to help treat and manage their obesity during pregnancy
- Women living with obesity who hold excess weight and are considering pregnancy receive dedicated maternal health counselling to help treat and manage their obesity during prior to an upcoming pregnancy

Columns:

- 0%
- 1– 10%
- 11- 20%
- 21-30%
- 31-40%
- 41-50%
- 51-60%
- 61-70%
- 71-80%
- 91-99%
- 100%
- Unsure

Q8. What services, if any, are available locally that people living with obesity can be referred to?

*matrix* Rows:

- Endocrinology
- Cardiology
- Nutrition/Dietetics
- Occupational therapy
- Behaviour/Lifestyle medicine
- Surgical obesity care (Metabolic/bariatric surgery)
- Gastroenterology
- General practice / Primary care providers
- Physical therapy
- Exercise physiology
- Obesity specialist centers
- Medical obesity treatment (anti-obesity medications)
- Mental health counselling
- Family counselling
- Home healthcare services
- Eating disorder treatment
- Alternative therapy (i.e., acupuncture, osteopathy etc.)
- Other services

Columns:

- Available and currently reimbursed
- Available but not currently reimbursed
- Not available/accessible to people living with obesity
- Unsure

**CANNOT SELECT 'Not available/accessible to people living with obesity' FOR ALL ROWS

Q9. What proportion of your patients living with obesity are referred to the following services to help manage their obesity? *matrix*

Rows:

- Endocrinology
- Cardiology
- Nutrition/Dietetics
- Occupational therapy
- Behavior/Lifestyle medicine
- Surgical obesity care (Metabolic/bariatric surgery)
- Gastroenterology
- General practice / Primary care providers
- Physical therapy
- Exercise physiology
- Obesity specialist centers
- Medical obesity treatment (anti-obesity medications)
- Mental health counselling
- Family counselling
- Home healthcare services
- Eating disorder treatment
- Alternative therapy (i.e., acupuncture, osteopathy etc.)
- Other services

Columns:

- 0%
- 1 – 10%
- 11- 20%
- 21-30%
- 31-40%
- 41-50%
- 51-60%
- 61-70%
- 71-80%
- 81-90%
- 91-99%
- 100%
- Unsure

Q10. Which of the following support methods are available in your department/country to help you deliver treatment and long-term management of people living with obesity *matrix*

Columns:

- Yes
- No
- Unsure
- I do not know

Rows:

- Frameworks that enable those supporting patients enough capacity (time) to care for number of patients with obesity
- Incentive structures for healthcare professionals (HCPs) in place which allow the prioritization, treatment and long-term management of people living with obesity
- Relevant resources (i.e., fact sheets, discussion guides, available online resources) for staff to enable them to deliver effective care
- Relevant resources (i.e., fact sheets, discussion guides, available online resources) for people living with obesity
- Telehealth consultations
- Digital health records enabling access to long-term patient health data
- Wearable technology available to provide to people living with / at risk of obesity to monitor their health
- Personalised digital health tools / mobile apps that enable people living with obesity to self- manage
- Sufficient clinical and social referral pathways / frameworks in place for people living with obesity
- Comprehensive diagnostic criteria (anthropometrics including measurement of weight circumference, biomarkers, medical history, psychosocial history, family history, physical exam etc.) are well defined
- Comprehensive screening process is in place
- Clinical tests to assess risk of obesity i.e., population testing for biomarkers of obesity

Q10b. Which of the following support methods are available in your department/country to help you deliver treatment and long-term management of people living with obesity *matrix*

Columns:

- Yes
- No
- Unsure
- I do not know

Rows:

- Pharmacy-based care enabling people living with obesity to obtain information about their obesity treatment, support with managing their disease and/or monitoring of signs and symptoms
- Community-based healthcare which can deliver care and support people living with obesity closer to their home
- Home healthcare services available to people living with obesity who are disabled by their disease
- Maternal health programs for women living with obesity who are pregnant or considering pregnancy
- Measures that allow for patient feedback/input into services and provision of care for obesity
- Information / training to raise awareness of weight bias and stigma and tackle misconceptions that may be preventing care
- Appropriate infrastructure/equipment (easy access to clinic or HCP, accommodation for people of different shapes and sizes (chairs, wheelchairs, beds, scales, cuffs etc.) to care for people living with obesity
- Increased options for continued professional development on obesity for HCPs
- Professional networks for those involved with obesity care

Q11. How would you rate the importance of the following measures to improve obesity care and management in your country? *MATRIX*

Columns:

- Very important,
- Somewhat important
- Somewhat unimportant
- Very unimportant
- Not sure

Rows:

- Government-level recognition of obesity as a chronic disease in plans, strategies and communication materials
- Official government categorization of obesity as a chronic disease in government communications
- Political commitment to deliver sustained action on obesity
- Well defined steps to manage obesity are in place
- National obesity plans and strategies for adults at risk or living with obesity
- National obesity plans and strategies for children at risk or living with obesity
- Supportive / adequate obesity policies
- Advertising controls or restrictions for high energy foods
- Legislation for front of package labelling that indicate products high in sugar, saturated fat, or energy
- Legislation for nutritional information (calories) to be visible on menus
- Taxes for high sugar products/high fat products
- Urban design which promotes physical activity and walking

Q11b. How would you rate the importance of the following measures to improve obesity care and management in your country? *MATRIX*

Columns:

- Very important,
- Somewhat important
- Somewhat unimportant
- Very unimportant
- Not sure

Rows:

- Comprehensive obesity guidelines (i.e., clinical practice guidelines)
- Sufficient clinical and social referral pathways / frameworks in place for people living with obesity
- Targeted interventions towards vulnerable populations requiring additional support due to the sociodemographic setting
- Targeted interventions to prevent or manage obesity in people with mental health conditions
- Targeted interventions to prevent or manage obesity in specific ethnic groups at higher risk of obesity
- Targeted interventions to support maternal health in people at risk or living with obesity
- Targeted interventions to address socio-economic inequalities in areas with high prevalence of obesity
- Awareness campaigns to educate the general public on the science of obesity
- Health promotion campaigns
- Environmental laws designed with obesity in mind to reduce chemical impact on endocrine disruptors
- School food and nutrition policies (including school food standards, food provision and nutrition education)
- Public institutions food and nutrition policies (including food standards, food provision and nutrition education)

Q11c. How would you rate the importance of the following measures to improve obesity care and management in your country? *MATRIX*

Columns:

- Very important
- Somewhat important
- Somewhat unimportant
- Very unimportant
- Not sure

Rows:

- Funding allocated specifically to surveillance of obesity
- Funding allocated specifically to support screening of obesity
- Funding allocated to improve the diagnosis of people living with obesity
- Funding allocated specifically to treatment of obesity
- Funding allocated to long-term management of obesity
- Funding allocated to deliver on clinical frameworks for obesity
- Funding allocated to public health interventions to enable healthier choices
- Funding allocated to generate ongoing data/evidence on impact of measures through monitoring and measurement
- Funding allocated to obesity research
- Incentives / measures to attract General Practitioners (GPs)/Primary care providers (PCPs) to ensure enough are available to deliver appropriate long-term care for people with obesity
- Incentives / measures to attract specialists (specialist GPs/PCPs, endocrinologist etc.) to ensure enough are available to provide care to people with obesity
- Incentives / measures to attract allied health providers (nurses, dietitians, psychologists,

social workers, kinesiologists, etc.) to ensure enough are available to provide care for people with obesity

Q11d. How would you rate the importance of the following measures to improve obesity care and management in your country? *MATRIX*

Columns:

- Very important
- Somewhat important
- Somewhat unimportant
- Very unimportant
- Not sure

Rows:

- Frameworks that enable those supporting patients enough capacity (time) to care for number of patients with obesity
- Incentive structures for healthcare professionals (HCPs) in place which allow the prioritization, treatment and long-term management of people living with obesity
- Relevant resources (i.e., factsheets, discussion guides, available online resources) for staff to enable them to deliver effective care
- Relevant resources (i.e., factsheets, discussion guides, available online resources) for people living with obesity
- Telehealth consultations
- Digital health records enabling access to long-term patient health data
- Wearable technology available to provide to people living with / at risk of obesity to monitor their health
- Personalised digital health tools / mobile apps that enable people living with obesity to self- manage
- Sufficient clinical and social referral pathways / frameworks in place for people living with obesity
- Comprehensive diagnostic criteria (anthropometrics including measurement of weight circumference, biomarkers, medical history, psychosocial history, family history, physical exam etc.) are well defined
- Comprehensive screening process is in place
- Clinical tests to assess risk of obesity i.e., population testing for biomarkers of obesity

Q11e . How would you rate the importance of the following measures to improve obesity care and management in your country? *MATRIX*

Columns:

- Very important
- Somewhat important
- Somewhat unimportant
- Very unimportant
- Not sure

Rows:

- Pharmacy-based care enabling people living with obesity to obtain information about their obesity treatment, support with managing their disease and/or monitoring of signs and symptoms
- Community-based healthcare which can deliver care and support people living with obesity closer to their home
- Home healthcare services available to people living with obesity who are disabled by their disease
- Maternal health programs for women living with obesity who are pregnant or considering pregnancy
- Measures that allow for patient feedback/input into services and provision of care for obesity
- Information / training to raise awareness of weight bias and stigma and tackle misconceptions that may be preventing care
- Appropriate infrastructure/equipment (easy access to clinic or HCP, accommodation for people of different shapes and sizes (chairs, wheelchairs, beds, scales, cuffs etc.) to care for people living with obesity
- Obesity education delivered within HCP academic training (i.e., medical schools, undergraduate/graduate specificalities, etc.)
- Increased options for continued professional development on obesity for HCPs
- Professional networks for those involved with obesity care
- Support from team, management, leadership to prioritize obesity

Q11f. How would you rate the importance of the following measures to improve obesity care and management in your country? *MATRIX*

Columns:

- Very important,
- Somewhat important
- Somewhat unimportant
- Very unimportant
- Not sure

Rows:

- Reimbursement of long-term specialist care (endocrinologist, cardiologist, obesity nurse specialist etc.)
- Reimbursement of allied health provider support (dieticians, psychologists, osteopaths etc.)
- Reimbursement of medical treatment
- Reimbursement of surgical treatment
- Insurance/finance plans for allied provider support (dieticians, psychologists, osteopaths etc.)
- Insurance/finance plans for counselling/psychological support
- Insurance/finance plans for obesity medication
- Insurance/finance plans for surgical treatment
- Insurance/finance plans for long-term specialist care (endocrinologist, cardiologist, obesity nurse specialist etc.)

Q12. In patients living with obesity would the following be offered as a first, second or final step / not at all? *MATRIX*

Rows:

- Ask permission to offer support
- Offer guidance on nutrition and physical activity
- Offer guidance on nutrition and physical activity and refer them to a dietician / exercise specialist
- Have a conversation with them to identify all factors that may be exacerbating signs and symptoms of their obesity (i.e., mental health, stress, poor sleep, medication, etc.)
- Guidance on relevant behavioral changes to implement to reduce exacerbating factors
- Referrals to relevant specialists based on factors identified in conversation
- Referral to relevant specialists to help identify causes
- Discuss treatment opportunities.
- Initiate treatment for all who meet the relevant BMI cut-off
- Initiate management (dedicated follow-ups) of people who present at risk of obesity
- Initiation of treatment based on comprehensive assessment of obesity indicators

Columns:

- Offered as a first step
- Offered as a second step
- Offered as final step
- Not offered at all
- I don’t know

Q13. What percentage of your patients living with obesity… *MATRIX*

Rows:

- Have regular consultations and follow-up with a general practitioner / primary care provider
- Are provided with education about the science of obesity including genetic, biological, environmental, and social determinants of health
- Are assessed for internalized weight bias
- Are provided with nutrition assessment/support by a professional (registered dietitian) with training in obesity care
- Are provided with physical activity assessment/support by a professional (exercise specialist/kinesiologist, etc.) with training in obesity care
- Are provided access to psychological/mental health assessment
- Are provided access to psychological/mental health support
- Are prescribed anti-obesity medications to manage obesity
- Of those on medication for other conditions, are offered a review of their medications
- Are provided access to long-term specialist care (endocrinologist, cardiologist, obesity nurse specialist etc.) to manage obesity
- Are referred for bariatric surgery
- Have undergone bariatric surgery

Columns:

- 0
- 1– 10%
- 11- 20%
- 21-30%
- 31-40%
- 41-50%
- 51-60%
- 61-70%
- 71-80%
- 81-90%
- 91-99%
- 100%

**CANNOT SELECT ‘0%’ FOR ALL ROWS**

Q14. What proportion of your patients living with obesity have comorbidities for which they receive treatment and/or specialist care? *matrix*

Rows:

- Cancer
- Diabetes
- Cardiovascular disease
- Gastrointestinal disorders
- Skin conditions
- Psychological (disordered eating patterns, mental health conditions, ADHD, etc.)
- Physical complications mobility limitations and /or complications
- Malnutrition (including micronutrients deficiencies, sarcopenic obesity, etc.)

Columns:

- 0%
- 1– 10%
- 11- 20%
- 21-30%
- 31-40%
- 41-50%
- 51-60%
- 61-70%
- 71-80%
- 81-90%
- 91-99%
- 100%
- Prefer not to say

**CANNOT SELECT ‘0%’ FOR ALL ROWS**

Q15. To what extent do you agree or disagree with the following statements? *matrix* Rows:

- Obesity is a result of personal and conscious decisions to perform a behavior that increase risk of obesity
- People are responsible for managing obesity on their own
- In my clinic, obesity care is well organized
- General practitioners (GPs)/ Primary Care Providers (PCPs) and specialists work well together to help people with obesity
- General Practitioners (GPs) / Primary Care Providers (PCPs) are mainly responsible to care for people with obesity
- Specialists are mainly responsible to care for people with obesity
- Allied health providers are mainly responsible to care for people with obesity
- There are enough General Practitioners (GPs) GPs/Primary care providers (PCPs) available to deliver appropriate long-term care for people with obesity
- There are enough specialists (specialist General Practitioners (GPs) / Primary Care Providers (PCPs), endocrinologist etc.) available to provide care to people with obesity
- There are enough allied health providers (nurses, dietitians, psychologists, social workers, kinesiologists, etc.) available to provide care for people with obesity
- There is sufficient capacity (time) available to those supporting patients to care for number of patients with obesity
- I get regular updates on new research within the obesity field

Columns

- Strongly agree
- Somewhat agree
- Neither agree nor disagree
- Somewhat disagree
- Strongly disagree

**CONTRADICTIONS**

CAN ‘Strongly agree’ OR ‘Somewhat agree’ TO ONE OF THESE ONLY:

General Practitioners (GPs) / Primary Care Providers (PCPs) are mainly responsible to care for people with obesity

Specialists are mainly responsible to care for people with obesity

Allied health providers are mainly responsible to care for people with obesity

Q16. To what extent do you agree or disagree with the following statements? *matrix* Rows:

- Obesity services are available to all people with obesity
- Obesity care needs more focus/funding/attention
- Obesity care is high on the healthcare priority
- Screening and early diagnosis of obesity is key to prevent new cases / progression of obesity
- More robust screening measures/methods are needed for obesity
- Referral pathways are clearly established
- Diagnostic criteria for obesity are clear and sufficient
- The facilities at the clinic/hospital are well equipped to welcome and manage people with obesity across all weights, shapes, and sizes

Columns

- Strongly agree
- Somewhat agree
- Neither agree nor disagree
- Somewhat disagree
- Strongly disagree

Q16b. To what extent do you agree or disagree with the following statements? *matrix*

Rows:

- There is a need for greater health literacy on obesity in the general public to inform decisions and actions towards people living with obesity
- There is a need for greater health literacy in people at risk of or living with obesity to inform decisions and actions regarding prevention, treatment, and management of obesity
- There is a need for greater health literacy on obesity in the medical community to inform decisions and actions regarding prevention, treatment, and management of people at risk or living with obesity
- I hold biases towards people with obesity
- My colleagues hold biases towards people with obesity
- People with obesity deserve the same respect, care, and treatment as all others with chronic diseases
- I feel well equipped to provide the best care for people living with obesity

Columns

- Strongly agree
- Somewhat agree
- Neither agree nor disagree
- Somewhat disagree
- Strongly disagree

Qi. Which best describes your healthcare coverage model?

- Universal healthcare (health care system provides coverage to a high percentage of citizens.)
- Public (free) health care (all citizens receive health care without having to pay for services)
- Private healthcare (all citizens pay for healthcare either directly or through private insurance schemes)

Qii. How many of the patients you see for obesity are in each of the following categories? *MATRIX* Rows:

- At risk
- Early stages
- Moderate obesity
- Severe obesity
- In remission
- Other

Columns:

- 0%
- 1-25%
- 26-50%
- 51-75%
- 76-100%

***CANNOT SELECT '0%' FOR ALL ROWS**

Qiii. How long have you been in your career?

- Under a year
- 1-3 years
- 4-5 years
- 6-8 years
- 9-10 years
- Over 10 years

**REGION AUSTRALIA**

Which region do you work in?

- New South Wales
- Northern Territory
- Queensland
- South Australia
- Tasmania
- Victoria
- Western Australia

# OPEN Models of Care Survey—Decision makers

Qa. In which one of the following industry sectors does your company operate in?

- Architecture, Engineering & Building
- Arts & Culture
- Education
- Finance
- Healthcare
- HR
- IT & Telecoms
- Legal
- Manufacturing & Utilities
- Retail, Catering & Leisure
- Sales, Media & Marketing
- Travel & Transport
- Other
- I do not work

**CONTINUE IF ‘Healthcare’ IS SELECTED**

Qb. How would you describe your role in your organisation/department decision making process for responsibility on implementation of obesity care?

- I have no input into the final decision [END]
- I provide input toward the final decision [END]
- I help reach the final decision as part of a group/committee
- I make the final decision with input from staff/management
- I am the sole decisionmaker

Qc. Which best describes you?

- I am a commissioner
- I am head of department
- I am head of a hospital, clinic or practice
- I am part of leadership team at a hospital, clinic or practice
- I sit on national / regional health committees
- I am a junior doctor (end)
- I work in administration (end)
- I work as an assistant or secretary (end)
- Other (end)

Q1. Which of the following, if any, best describes how you would categorise obesity?

(Select 1 option)

- A lifestyle condition (reversable condition which is a result of poor habitual and active personal choices made by the individual)
- A circumstantial condition (reversable condition caused by the circumstances

surrounding a person i.e., low socio-economic status, food deserts, lack of green spaces etc.)

- A condition (a reversable condition caused by general poor health)
- A disease (a temporary disease caused by a multitude of factors (physiological, genetic, environmental etc.), with little or no outstanding or lasting effects once treated
- A chronic disease (caused by a multitude of factors (physiological, genetic, environmental etc.) lasting 1 year or more and requiring ongoing management following remission
- I am not sure
- None of the above

Q2. How confident, if at all, do you feel in your understanding of obesity as a chronic disease? (Consider things like the clinical definition (defined by excess and/or abnormal adipose tissue that impairs health), disease progression, triggers, and treatment needs)

- 1 – very confident
- 2 – somewhat confident
- 3 – neither confident nor unconfident
- 4 – not very confident
- 5 – not confident at all

Q3. When considering funding allocation, how high of a priority, if at all, would you assess the following to be in your practice, institution, department, commissioning group etc.? *matrix*

Rows:

- Primary prevention
- Secondary prevention
- Tertiary prevention
- Screening and early diagnosis
- Diagnosis
- Treatment
- Long-term management
- Self-management

Columns:

- Top priority
- Within top five priorities
- Within top 6-10 priorities
- Not a priority

Q4. Approximately what percentage of your resources (practice, institution, department, commissioning group etc.) are allocated to areas within obesity? *matrix*

Rows:

- Research
- Data collection
- HCP training
- Patient support programs
- Maternal health programs specific to pregnant women
- Policy work (advisory / health committees, panels, advocacy)
- Engagement with patient and HCP organizations
- Equipment (blood pressure cuffs, scales etc.)
- Infrastructure (chairs, beds, wheelchairs, easy access to rooms etc.)
- Other areas

Columns:

- 0%
- 1– 10%
- 11- 20%
- 21-30%
- 31-40%
- 41-50%
- 51-60%
- 61-70%
- 71-80%
- 81-90%
- 91-99%
- 100%
- I don’t know /am not sure

Q5. Are there dedicated/specific obesity clinical practice guidelines (CPGs) (as opposed to guidelines on obesity included within guidelines for other conditions, i.e., diabetes) sanctioned for use in your health system or country? (Select 1 option)

- Yes, we have dedicated CPGs for obesity, which we regularly review when assessing obesity services
- Yes, we have dedicated CPGs for obesity, but find them inadequate
- Yes, we have dedicated CPGs for obesity, but we rarely consult them when assessing obesity services
- Yes, we have dedicated clinical guidelines CPGs for obesity, but we do not have the resources to implement them or the services to refer patients to them (e.g., surgery, psychologist)
- Yes, other please specify
- No, we do not have dedicated obesity guidelines
- Not applicable, we follow guidelines from a different disease area that has obesity guidance
- Not applicable, we have made our own guidelines
- I am unaware if we have or follow any CPGs for obesity

Q6. Do any guidelines on obesity (dedicated or as part of guidelines for other diseases) cover the following? (Tick all that apply)

- Risk assessment/ Screening of obesity indicators
- Early diagnosis
- Diagnosis
- Disease progression
- Treatment
- Long-term management
- Nutrition
- Physical activity
- Behavioral therapies
- Mental health counseling
- Pharmacotherapy
- Surgical interventions
- None of the above *exclusive*

Q7. Thinking specifically about people with obesity, who, if any of the below are healthcare professionals financially incentivized to treat obesity in? (Select all that apply)

- People of non-white ethnicities diagnosed with obesity based on ethnicity-specific BMI cutoffs 30 kg/m2 without comorbidities
- People of non-white ethnicities diagnosed with obesity based on ethnicity-specific BMI cutoffs 30 kg/m2 with comorbidities
- People with BMI between 30 -34 without comorbidities
- People with BMI between 30-34 with comorbidities.
- People with BMI over 35 without comorbidities
- People with BMI over 35 with comorbidities
- People identified as being at risk of obesity based on comprehensive screening of obesity indicators (not specific to BMI)
- People diagnosed with obesity based on comprehensive diagnostic criteria (not specific to BMI)
- People living with obesity when deemed a risk factor for other conditions
- None of the above *exclusive*
- I am not sure/I do not know *exclusive*

Q8. What services, if any, are available locally that people living with obesity can be referred to?

*matrix* Rows:

- Endocrinology
- Cardiology
- Nutrition/Dietetics
- Occupational therapy
- Behaviour/Lifestyle medicine
- Surgical obesity care (Metabolic/bariatric surgery)
- Gastroenterology
- General practice / Primary care providers
- Physical therapy
- Exercise physiology
- Obesity specialist centers
- Medical obesity treatment (anti-obesity medications)
- Mental health counselling
- Family counselling
- Home healthcare services
- Eating disorder treatment
- Alternative therapy (i.e., acupuncture, osteopathy etc.)
- Other services

Columns:

- Available and currently reimbursed
- Available but not currently reimbursed
- Not available/accessible to people living with obesity
- Unsure

Q9. What have you (re: your practice, institution, department, commissioning group etc.) set out as the standard of care for people living with obesity as a first, second and final step? *MATRIX*

Rows:

- Diagnosing obesity in a healthcare setting
- Guidance on nutrition and physical activity
- Guidance on nutrition and physical activity and referral to a dietician / exercise specialist
- Review of factors that may be exacerbating signs and symptoms of their obesity (i.e., mental health, stress, poor sleep, medication, nutrition, physical activity, trauma/abuse, social determinants of health etc.)
- Guidance on relevant behavioral changes to implement to reduce exacerbating factors
- Referrals to relevant specialists based on factors identified in conversation
- Referral to relevant specialists to help identify causes and manage disease
- Review of surgical/pharmacological treatment opportunities
- Initiation of treatment of people who have a BMI at 30 kg/m2 or above with no comorbidities
- Initiation of treatment of people with established BMI cut-off with comorbidities
- Initiation of treatment of people with established BMI cut-off without comorbidities
- Initiation of treatment based on comprehensive assessment of obesity indicators
- Initiate management (dedicated follow-ups) of people who present at risk of obesity
- Columns:
- Offered as a first step
- Offered as a second step
- Offered as a final step
- Not offered at all
- I don’t know
- Separate opt out: There is not set standard of care established

Q10. Which of the following measures, if any, has the government/health authorities, to your knowledge, set out (strategies, plans, frameworks, guidance) or incentivized (funded) in efforts to prevent and manage obesity? (Choose all that apply)

- Government-level recognition of obesity as a chronic disease in plans, strategies and communication materials
- Official government categorization of obesity as a chronic disease in government communications
- Political commitment to deliver sustained action on obesity
- National obesity plans and strategies for adults at risk or living with obesity
- National obesity plans and strategies for children at risk or living with obesity
- Advertising controls or restrictions for high energy foods
- Legislation for front of package labelling that indicate products high in sugar, saturated fat, or energy
- Legislation for nutritional information (calories) to be visible on menus
- Taxes for high sugar products/high fat products
- Urban design which promotes physical activity and walking
- None of these *exclusive*
- I do not know about ANY measures the government has set out or incentivized

*exclusive* [SKIP Q11/Q12]

Q11. Which of the following measures, if any, has the government/health authorities, to your knowledge, set out (strategies, plans, frameworks, guidance) or incentivized (funded) in efforts to prevent and manage obesity? (Choose all that apply)

- Targeted interventions towards vulnerable populations requiring additional support due to their sociodemographic setting
- Targeted interventions to prevent or manage obesity in people with mental health conditions
- Targeted interventions to prevent or manage obesity in specific ethnic groups at higher risk of obesity
- Targeted interventions to support maternal health in people at risk or living with obesity
- Targeted interventions to address socio-economic inequalities in areas with high prevalence of obesity
- Awareness campaigns to educate the general public on the science of obesity
- Health promotion campaigns
- Environmental laws designed with obesity in mind to reduce chemical impact on endocrine disruptors
- School food and nutrition policies (including school food standards, food provision and nutrition education)
- Public institutions food and nutrition policies (including food standards, food provision and nutrition education)
- None of these *exclusive*

Q12. Which of the following measures, if any, has the government/health authorities, to your knowledge, set out (strategies, plans, frameworks, guidance) or incentivized (funded) in efforts to prevent and manage obesity? (Choose all that apply)

- Funding allocated specifically to surveillance of obesity
- Funding allocated specifically to support screening of obesity
- Funding allocated to improve the diagnosis of people living with obesity
- Funding allocated specifically to treatment of obesity
- Funding allocated to long-term management of obesity
- Funding allocated to deliver on clinical frameworks for obesity
- Funding allocated to public health interventions to enable healthier choices
- Funding allocated to generate ongoing data/evidence on impact of measures through monitoring and measurement
- Funding allocated to obesity research
- Incentives / measures to attract General Practitioners (GPs)/Primary care providers

(PCPs) to ensure enough are available to deliver appropriate long-term care for people with obesity

- Incentives / measures to attract specialists (specialist GPs/PCPs, endocrinologist etc.) to ensure enough are available to provide care to people with obesity
- Incentives / measures to attract allied health providers (nurses, dietitians, psychologists, social workers, kinesiologists, etc.) to ensure enough are available to provide care for people with obesity
- None of these *exclusive*

Q13. Which of the following financial support, if any, is available to people living with obesity? (Choose all that apply)

- Insurance/finance plans for allied provider support (dieticians, psychologists, osteopaths etc.)
- Insurance/finance plans for counselling/psychological support
- Insurance/finance plans for obesity medication
- Insurance/finance plans for surgical treatment
- Insurance/finance plans for long-term specialist care (endocrinologist, cardiologist, obesity nurse specialist etc.)
- None of the above *exclusive*
- I don’t know / am unsure *exclusive*

Q14. How would you rate the importance of the following measures to improve obesity care and management in your country? *MATRIX*

Columns:

- Very important
- Somewhat important
- Somewhat unimportant
- Very unimportant
- Not sure

Rows:

- Government-level recognition of obesity as a chronic disease in plans, strategies and communication materials
- Official government categorization of obesity as a chronic disease in government communications
- Political commitment to deliver sustained action on obesity
- Well defined steps to manage obesity are in place
- National obesity plans and strategies for adults at risk or living with obesity
- National obesity plans and strategies for children at risk or living with obesity
- Supportive / adequate obesity policies
- Advertising controls or restrictions for high energy foods
- Legislation for front of package labelling that indicate products high in sugar, saturated fat, or energy
- Legislation for nutritional information (calories) to be visible on menus
- Taxes for high sugar products/high fat products
- Urban design which promotes physical activity and walking

Q14b. How would you rate the importance of the following measures to improve obesity care and management in your country? *MATRIX*

Columns:

- Very important
- Somewhat important
- Somewhat unimportant
- Very unimportant
- Not sure

Rows:

- Comprehensive obesity guidelines (i.e., clinical practice guidelines)
- Sufficient clinical and social referral pathways / frameworks in place for people living with obesity
- Targeted interventions towards vulnerable populations requiring additional support due to the sociodemographic setting
- Targeted interventions to prevent or manage obesity in people with mental health conditions
- Targeted interventions to prevent or manage obesity in specific ethnic groups at higher risk of obesity
- Targeted interventions to support maternal health in people at risk or living with obesity
- Targeted interventions to address socio-economic inequalities in areas with high prevalence of obesity
- Awareness campaigns to educate the general public on the science of obesity
- Health promotion campaigns
- Environmental laws designed with obesity in mind to reduce chemical impact on endocrine disruptors
- School food and nutrition policies (including school food standards, food provision and nutrition education)
- Public institutions food and nutrition policies (including food standards, food provision and nutrition education)

Q14c. How would you rate the importance of the following measures to improve obesity care and management in your country? *MATRIX*

Columns:

- Very important
- Somewhat important
- Somewhat unimportant
- Very unimportant
- Not sure

Rows:

- Funding allocated specifically to surveillance of obesity
- Funding allocated specifically to support screening of obesity
- Funding allocated to improve the diagnosis of people living with obesity
- Funding allocated specifically to treatment of obesity
- Funding allocated to long-term management of obesity
- Funding allocated to deliver on clinical frameworks for obesity
- Funding allocated to public health interventions to enable healthier choices
- Funding allocated to generate ongoing data/evidence on impact of measures through monitoring and measurement
- Funding allocated to obesity research
- Incentives / measures to attract General Practitioners (GPs)/Primary care providers (PCPs) to ensure enough are available to deliver appropriate long-term care for people with obesity
- Incentives / measures to attract specialists (specialist GPs/PCPs, endocrinologist etc.) to ensure enough are available to provide care to people with obesity
- Incentives / measures to attract allied health providers (nurses, dietitians, psychologists, social workers, kinesiologists, etc.) to ensure enough are available to provide care for people with obesity

Q14d. How would you rate the importance of the following measures to improve obesity care and management in your country? *MATRIX*

Columns:

- Very important
- Somewhat important
- Somewhat unimportant
- Very unimportant
- Not sure

Rows:

- Frameworks that enable those supporting patients enough capacity (time) to care for number of patients with obesity
- Incentive structures for healthcare professionals (HCPs) in place which allow the prioritization, treatment and long-term management of people living with obesity
- Relevant resources (i.e., factsheets, discussion guides, available online resources) for staff to enable them to deliver effective care
- Relevant resources (i.e., factsheets, discussion guides, available online resources) for people living with obesity
- Telehealth consultations
- Digital health records enabling access to long-term patient health data
- Wearable technology available to provide to people living with / at risk of obesity to monitor their health
- Personalised digital health tools / mobile apps that enable people living with obesity to self-manage
- Sufficient clinical and social referral pathways / frameworks in place for people living with obesity
- Comprehensive diagnostic criteria (anthropometrics including measurement of weight circumference, biomarkers, medical history, psychosocial history, family history, physical exam etc.) are well defined
- Comprehensive screening process is in place
- Clinical tests to assess risk of obesity i.e., population testing for biomarkers of obesity

Q14e. How would you rate the importance of the following measures to improve obesity care and management in your country? *MATRIX*

Columns:

- Very important
- Somewhat important
- Somewhat unimportant
- Very unimportant
- Not sure

Rows:

- Pharmacy-based care enabling people living with obesity to obtain information about

their obesity treatment, support with managing their disease and/or monitoring of signs and symptoms

- Community-based healthcare which can deliver care and support people living with obesity closer to their home
- Home healthcare services available to people living with obesity who are disabled by their disease
- Maternal health programs for women living with obesity who are pregnant or considering pregnancy
- Measures that allow for patient feedback/input into services and provision of care for obesity
- Information / training to raise awareness of weight bias and stigma and tackle misconceptions that may be preventing care
- Appropriate infrastructure/equipment (easy access to clinic or HCP, accommodation for people of different shapes and sizes (chairs, wheelchairs, beds, scales, cuffs etc.) to care for people living with obesity
- Obesity education delivered within HCP academic training (i.e., medical schools, undergraduate/graduate specificalities, etc.)
- Increased options for continued professional development on obesity for HCPs
- Professional networks for those involved with obesity care
- Support from team, management, leadership to prioritize obesity

Q14f. How would you rate the importance of the following measures to improve obesity care and management in your country? *MATRIX*

Columns:

- Very important
- Somewhat important
- Somewhat unimportant
- Very unimportant
- Not sure

Rows:

- Reimbursement of long-term specialist care (endocrinologist, cardiologist, obesity nurse specialist etc.)
- Reimbursement of allied health provider support (dieticians, psychologists, osteopaths etc.)
- Reimbursement of medical treatment
- Reimbursement of surgical treatment
- Insurance/finance plans for allied provider support (dieticians, psychologists, osteopaths etc.)
- Insurance/finance plans for counselling/psychological support
- Insurance/finance plans for obesity medication
- Insurance/finance plans for surgical treatment
- Insurance/finance plans for long-term specialist care (endocrinologist, cardiologist, obesity nurse specialist etc.)

Caveat Q15: If you answer an open response, please do it in English language.

**BOLD/UNDERLINE 'Engllish language'***

Q15. What factors weigh the most in your decisions when forming obesity plans/guidance in your area/practice/department? Tick up to 3.

- National chronic disease guidelines
- Political focus
- Guidance from key national health authorities
- Guidance from key international authorities
- Guidance from medical associations
- Resource availability
- Number of people with obesity presenting in your health service / area
- Patient groups
- I consult HCPs in my clinic/area
- Medical data and research within obesity
- Other, please specify
- No factors in particular weigh the most *exclusive*

Q16. To what extent do you agree or disagree with the following statements: *MATRIX* Rows

- Obesity is a result of personal and conscious decisions to perform a behavior that increase risk of obesity
- People are responsible for managing obesity on their own
- Obesity care is well organized across health services
- General practitioners (GPs)/ primary care providers (PCPs) and specialists work well together to help people with obesity
- General practitioners (GPs)/primary care providers (PCPs) are mainly responsible to care for people with obesity
- Specialists are mainly responsible to care for people with obesity
- Allied health providers are mainly responsible to care for people with obesity
- There are enough General practitioners (GPs)Primary care providers (PCPs) available to deliver appropriate long-term care for people with obesity
- There are enough specialists (specialist General practitioners (GPs)/primary care providers (PCPs), endocrinologist etc.) available to provide care to people with obesity
- There are enough allied health providers (nurses, dietitians, psychologists, social workers, kinesiologists, etc.) available to provide care for people with obesity
- There is sufficient capacity (time) available to those supporting patients to care for number of patients with obesity
- I get regular updates on the new research within obesity
- Obesity services are available to all people with obesity
- Obesity care needs more focus/funding/attention
- Obesity care is high on the healthcare priority

Columns

- Strongly agree
- Somewhat agree
- Neither agree nor disagree
- Somewhat disagree
- Strongly disagree

**CONTRADICTIONS**

CAN ‘Strongly agree’ OR ‘Somewhat agree’ TO ONE OF THESE ONLY:

- GPs/PCPs are mainly responsible to care for people with obesity
- Specialists are mainly responsible to care for people with obesity
- Allied health providers are mainly responsible to care for people with obesity

Q17. To what extent do you agree or disagree with the following statements?: *MATRIX* Rows

- Screening and early diagnosis of obesity is key to prevent new cases / progression of obesity
- More robust screening measures/methods are needed for obesity
- Referral pathways are clearly established
- Diagnostic criteria for obesity are clear and sufficient
- The facilities at clinical/health settings (i.e., clinics, health centers, hospitals etc.) are well equipped to welcome and manage people with obesity
- There is a need for greater health literacy on obesity in the general public to inform decisions and actions towards people living with obesity
- There is a need for greater health literacy in people at risk of or living with obesity to inform decisions and actions regarding prevention, treatment, and management of obesity
- There is a need for greater health literacy on obesity in the medical community to inform decisions and actions regarding prevention, treatment, and management of people at risk or living with obesity
- I hold biases towards people with obesity
- My colleagues hold biases towards people with obesity
- People with obesity deserve the same respect, care, and treatment as all others with chronic diseases
- I feel well equipped to deliver the best care for people living with obesity

Columns

- Strongly agree
- Somewhat agree
- Neither agree nor disagree
- Somewhat disagree
- Strongly disagree

Qi. Which best describes your healthcare coverage model?

- Universal healthcare (health care system provides coverage to a high percentage of citizens.)
- Public (free) health care (all citizens receive health care without having to pay for services)
- Private healthcare (all citizens pay for healthcare either directly or through private insurance schemes)

Qii. When defining care for people living with obesity, how highly do you consider the different patient groups? *MATRIX*

Rows:

- At risk of obesity
- Early stages of obesity
- Moderate obesity
- Severe obesity
- In remission from obesity

Columns:

- High consideration of care
- Medium consideration of care
- Low consideration of care
- No consideration of care

Qiii. How long have you been in your career?

- Under a year
- 1-3 years
- 4-5 years
- 6-8 years
- 9-10 years
- Over 10 years

**REGION AUSTRALIA**

Which region do you work in?

- New South Wales
- Northern Territory
- Queensland
- South Australia
- Tasmania
- Victoria
- Western Australia

# **Table S1.** Demographic characteristics of survey population.

|  | **Overall** | **Australia** | **Brazil** | **Canada** | **Germany** | **Italy** | **Malaysia** | **Spain** | **Turkey** |
| --- | --- | --- | --- | --- | --- | --- | --- | --- | --- |
| **HCPs** |  |  |  |  |  |  |  |  |  |
| Age (years) |  |  |  |  |  |  |  |  |  |
| Mean | 37.7 | 36.3 | 36 | 37 | 39.1 | 40.6 | 36 | 39.7 | 36.7 |
| 22-30 years (%) | 27.6 | 27 | 27 | 27.8 | 27.5 | 28.7 | 28.7 | 27.7 | 27.3 |
| 31-40 years (%) | 35.4 | 35.8 | 34.9 | 35.4 | 35.2 | 35.4 | 35.6 | 36 | 35.3 |
| 41-50 years (%) | 44.4 | 44.4 | 44.1 | 44.5 | 43.7 | 44.4 | 44.3 | 44.7 | 44.8 |
| ≥51 years (%) | 57.2 | 61.2 | 56.8 | 56.1 | 55.8 | 60 | 51 | 57.7 | 53.7 |
| Specialty |  |  |  |  |  |  |  |  |  |
| Mean hours of medical education training on obesity (any specialty) | 14.2 | 14.2 | 15.7 | 14.8 | 12.6 | 12.0 | 12.6 | 16.0 | 15.6 |
| Mean hours of postgraduate training on obesity (any specialty) | 14.3 | 14.1 | 15.0 | 14.8 | 13.6 | 12.6 | 13.0 | 15.9 | 15.2 |
| GP/PCP |  |  |  |  |  |  |  |  |  |
| Mean years in practice | 7.7 | 7.5 | 7.3 | 8.2 | 6.7 | 7.6 | 8.2 | 8.2 | 7.9 |
| Mean hours postgraduate training on obesity | 14.5 | 13.4 | 15 | 15.9 | 13.9 | 12.7 | 14 | 16.1 | 15.2 |
| Endocrinologist |  |  |  |  |  |  |  |  |  |
| Mean years in practice | 7.3 | 7 | 7.1 | 7.7 | 7.4 | 7.1 | 6.7 | 9 | 6.9 |
| Mean hours postgraduate training on obesity | 14.7 | 14.6 | 18.4 | 14.2 | 15 | 13.4 | 11.1 | 16.9 | 15.7 |
| Cardiologist |  |  |  |  |  |  |  |  |  |
| Mean years in practice | 7.4 | 6.8 | 7.5 | 7.5 | 8.2 | 7.1 | 7.1 | 8.2 | 7.2 |
| Mean hours postgraduate training on obesity | 14.7 | 14.8 | 15.2 | 16.4 | 14.5 | 11.3 | 14.3 | 17.8 | 14.2 |
| Practice nurse |  |  |  |  |  |  |  |  |  |
| Mean years in practice | 6.6 | 6 | 6.3 | 6.4 | 7 | 7.5 | 5.9 | 7.4 | 6.2 |
| Mean hours postgraduate training on obesity | 13.2 | 13.5 | 12.7 | 12.6 | 10.1 | 13.1 | 12.6 | 14 | 15.8 |
| **HC DMs** |  |  |  |  |  |  |  |  |  |
| Head of organization/ institution |  |  |  |  |  |  |  |  |  |
| Mean years in career | 7.5 | 7.2 | 7.6 | 7.5 | 8.3 | 6.8 | 6.8 | 8.4 | 7.6 |
| Serve on national/ regional health committee |  |  |  |  |  |  |  |  |  |
| Mean years in career | 6.0 | 7.0 | 2.3 | 3.3 | 5.8 | 4.9 | 4.5 | 8.2 | 9.0 |

GP/PCP, general practitioner/primary care provider; HCP, healthcare practitioner; HC DM, healthcare decision maker.

# **Figure S1.** Healthcare practitioner (HCP) responses to: “Qii. What proportion of your patients living with obesity have comorbidities for which they receive treatment and/or specialist care?”


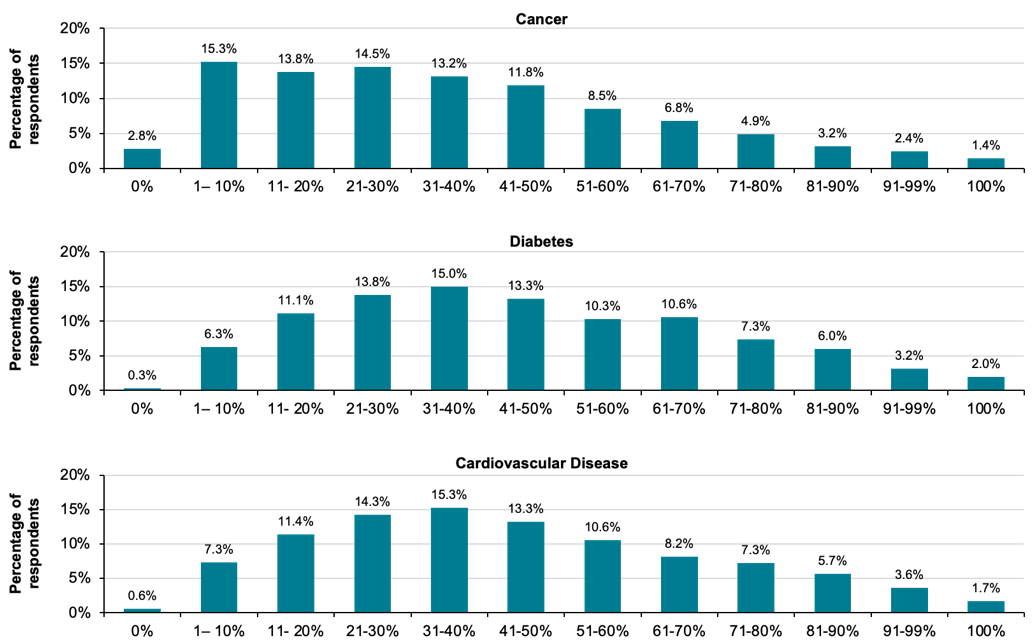


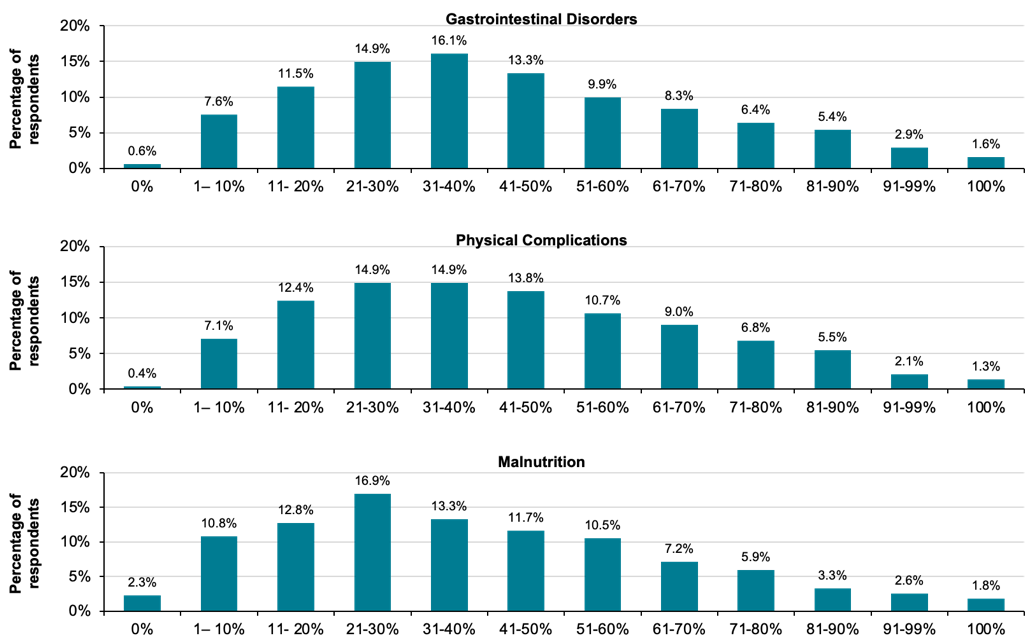


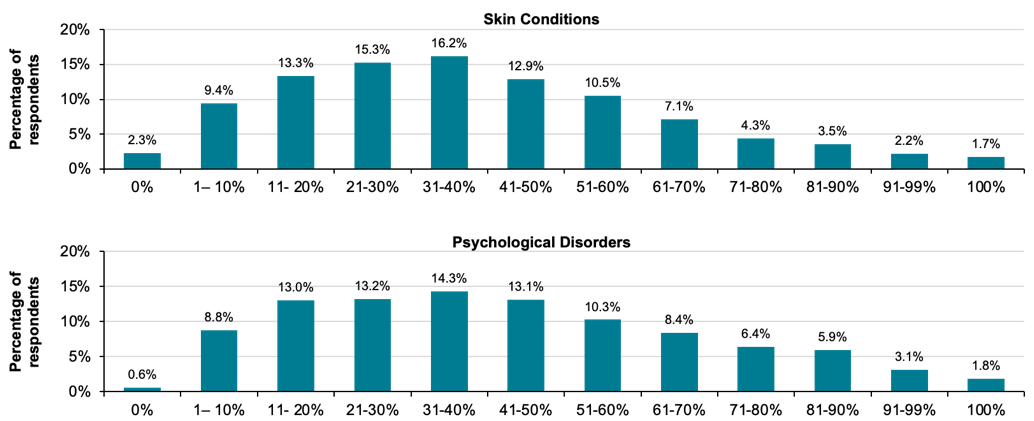


# **Figure S2.** Healthcare practitioner (HCP) responses to: “Q15. To what extent do you agree or disagree with the following statements?” with regard to statements about HCP attitudes about obesity.


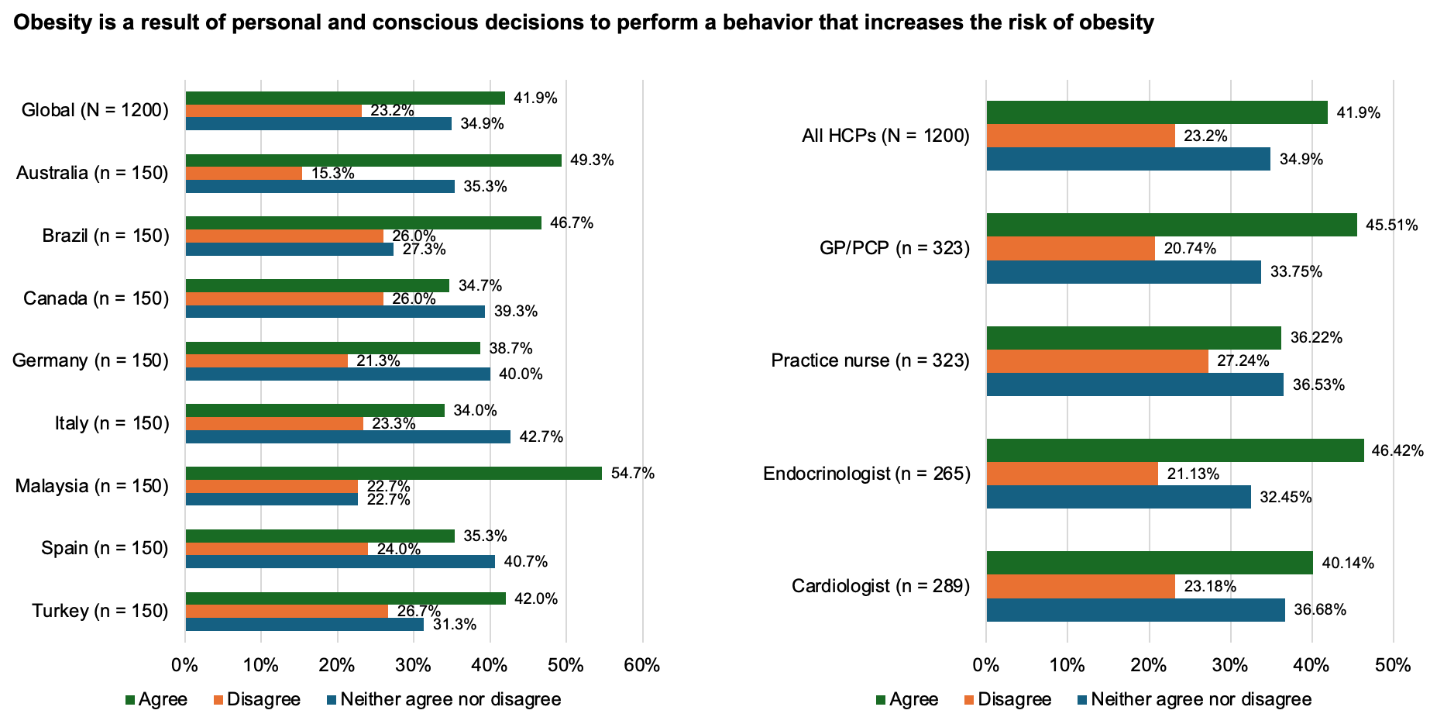


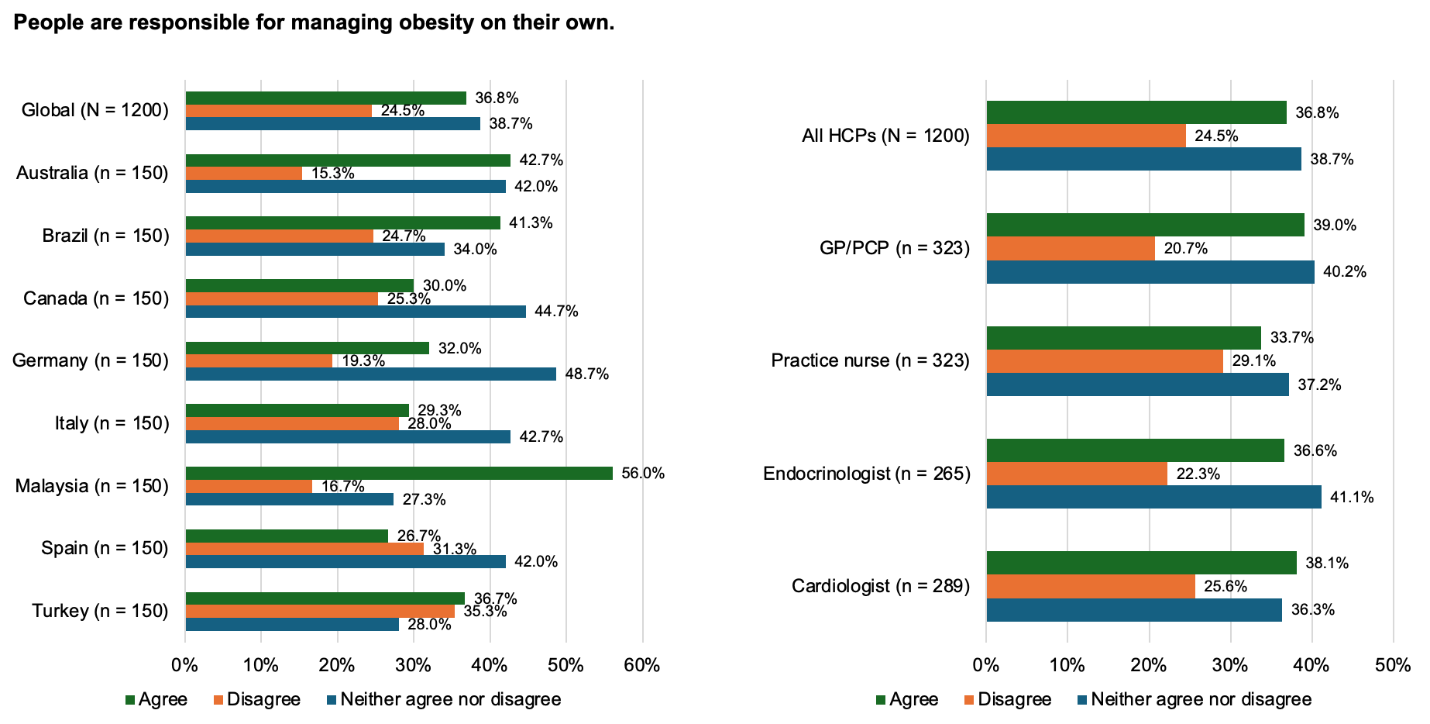


# **Figure S3.** Use of clinical practice guidelines (CPG)s.

(A, B) Healthcare practitioner (HCP) responses by specialty (A) and country (B) regarding use of clinical practice guidelines (CPGs) for obesity. (C, D) Healthcare decision maker (HC DM) responses regarding use of CPGs (C) and content of CPGs (D).


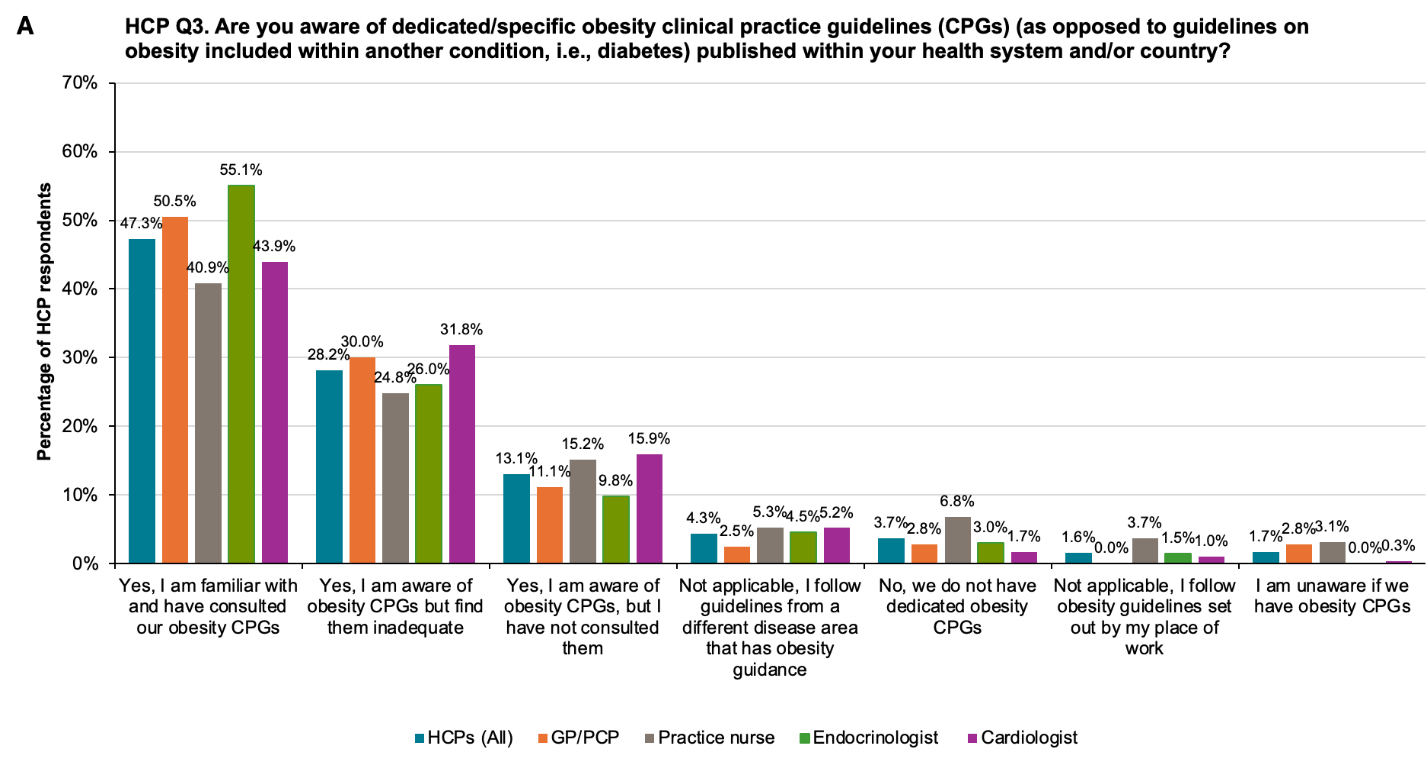


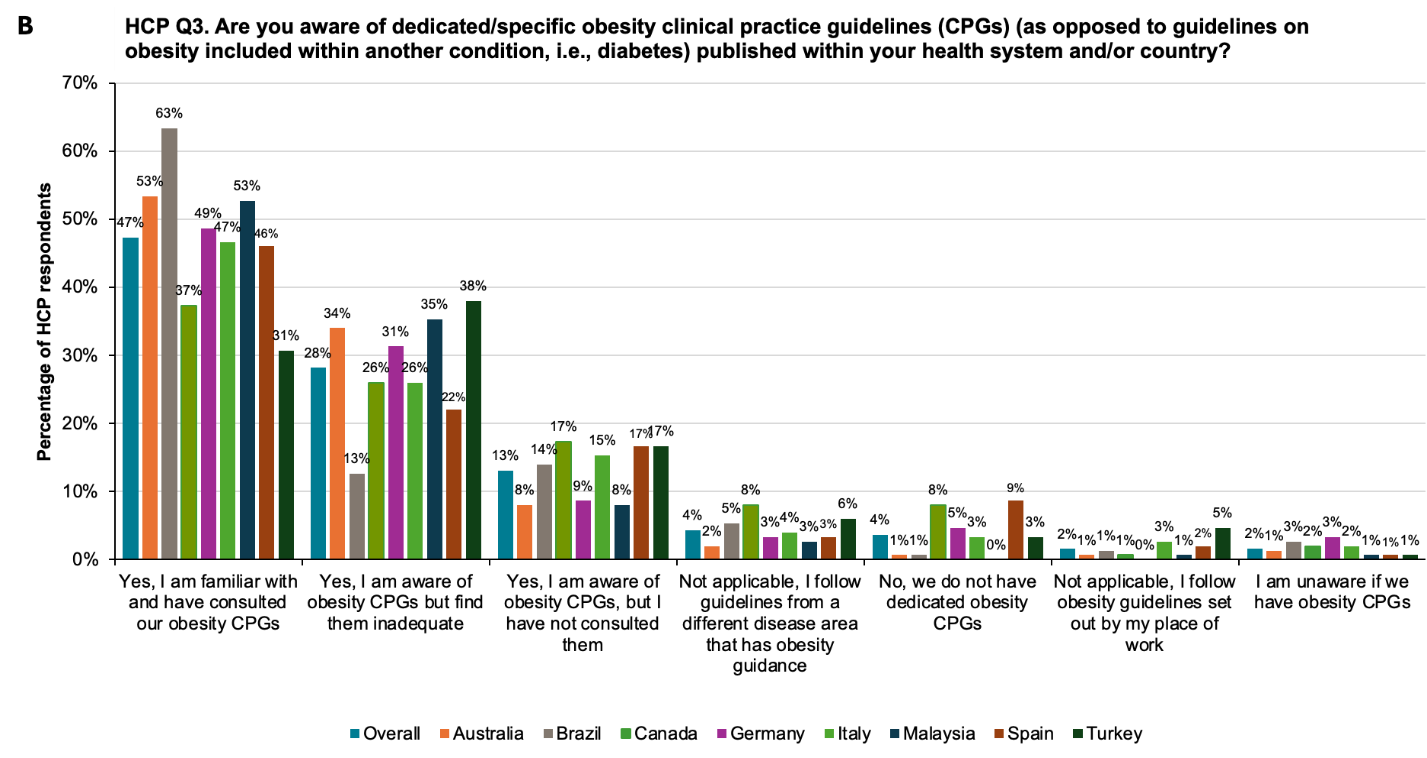


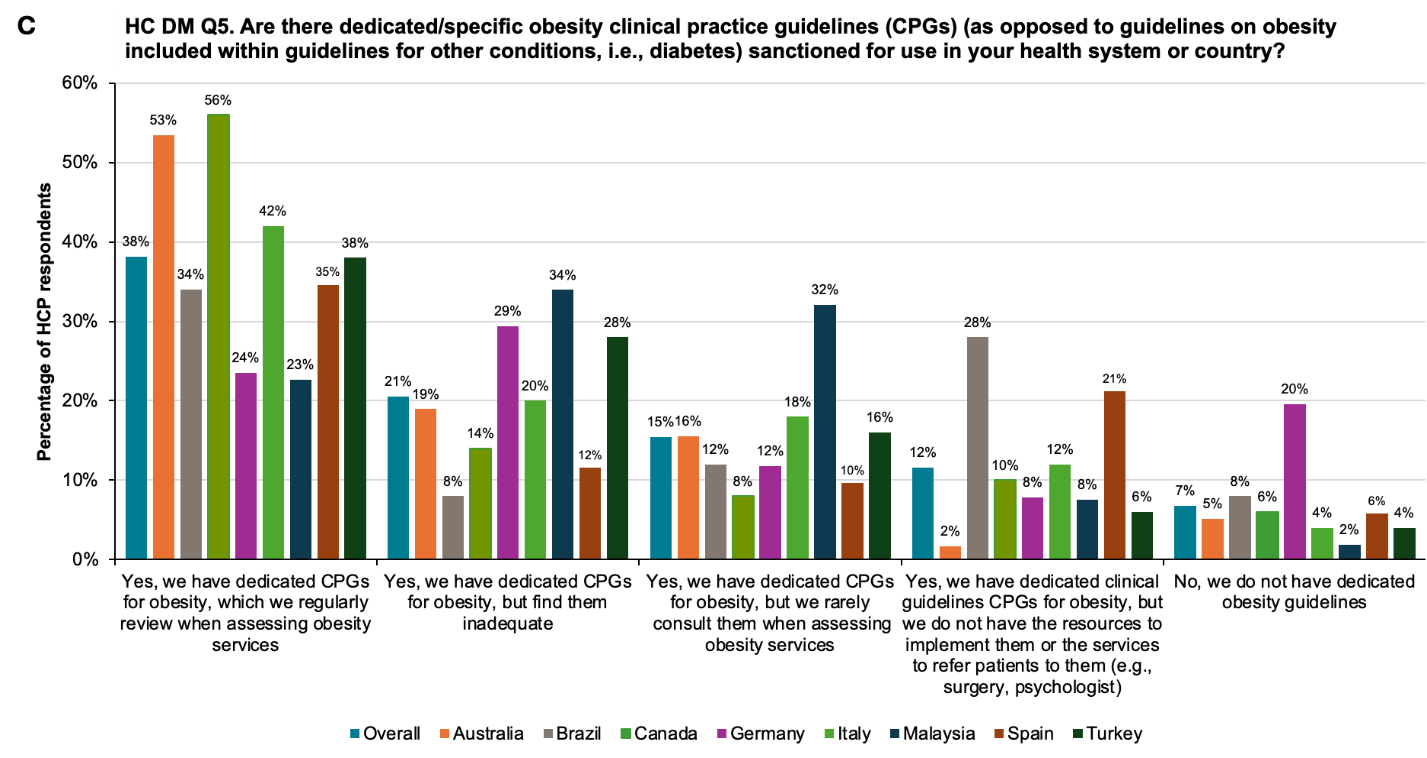


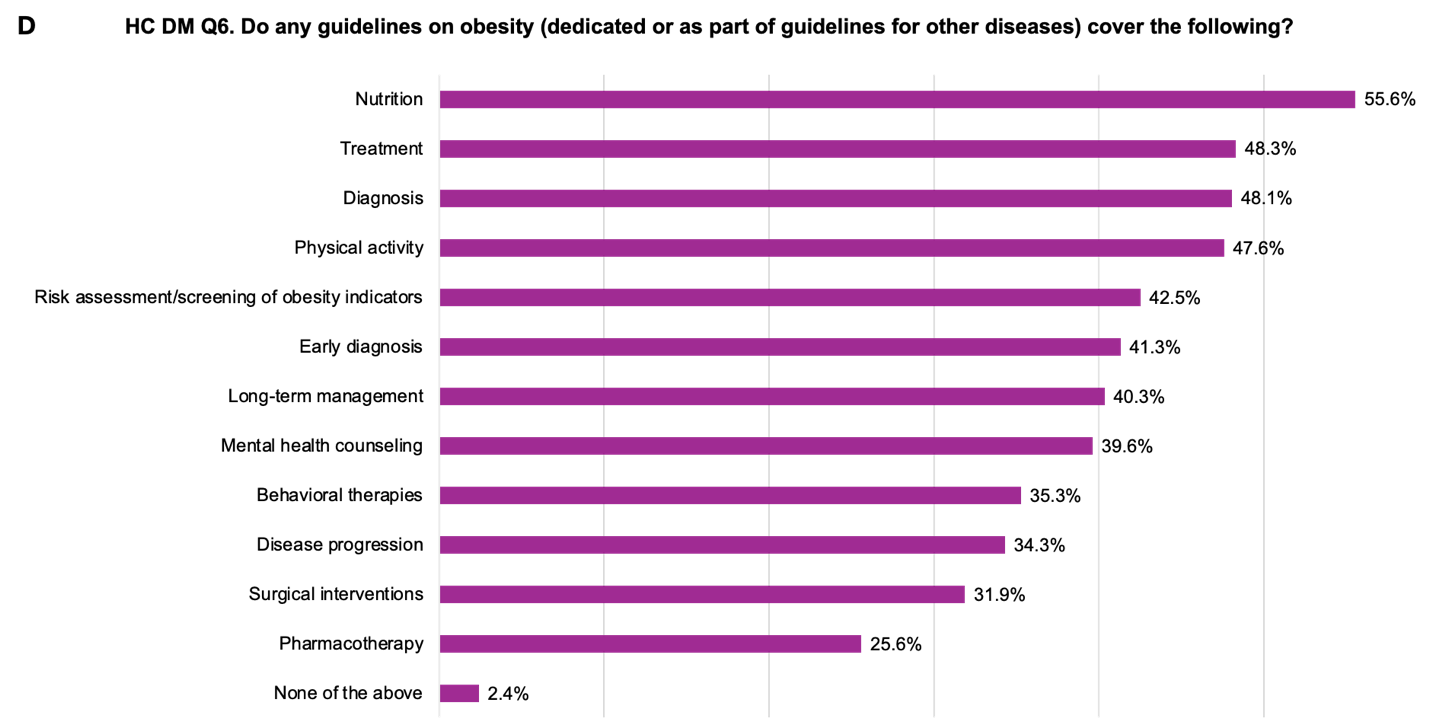


# **Figure S4.** Healthcare practitioner (HCP)–reported methods of assessing whether patients have obesity by specialty (A) and country (B).


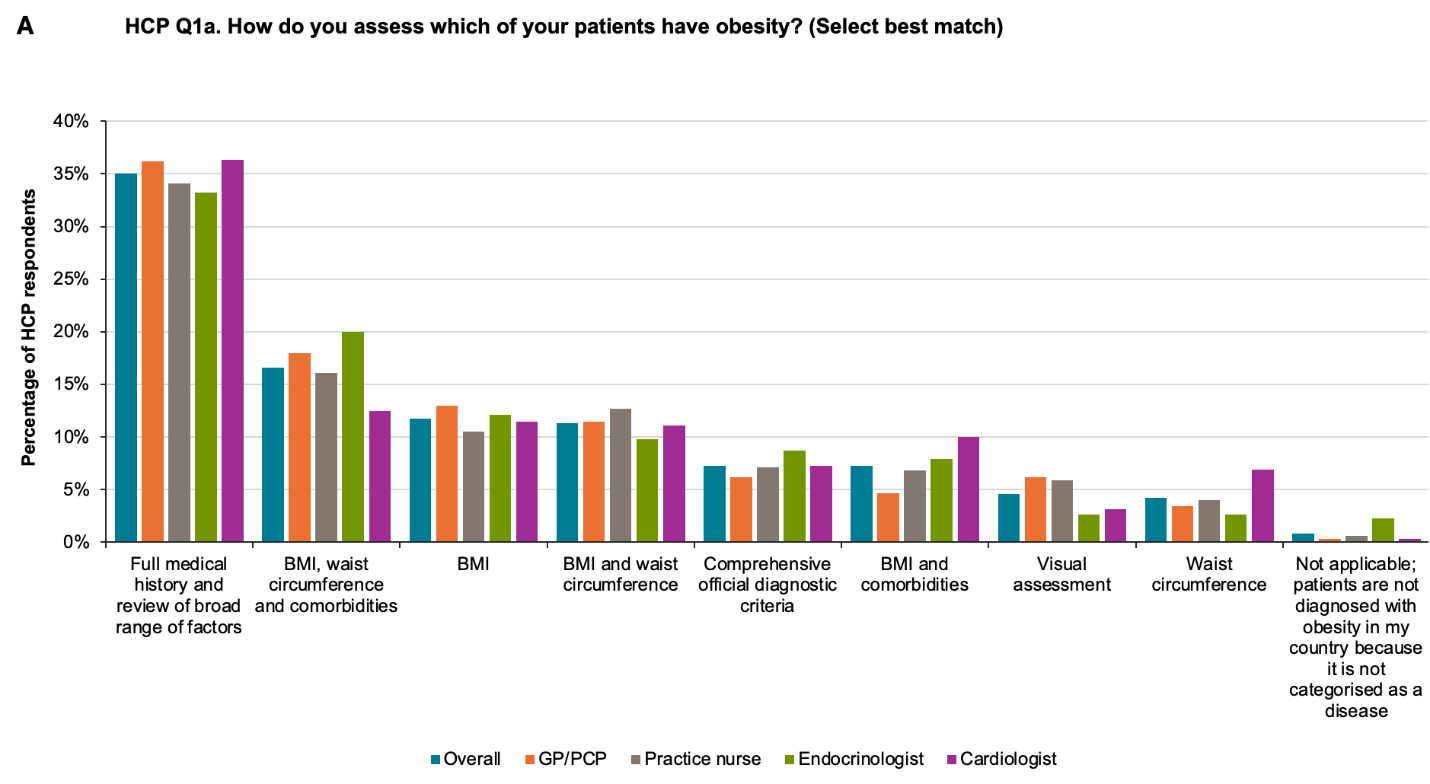


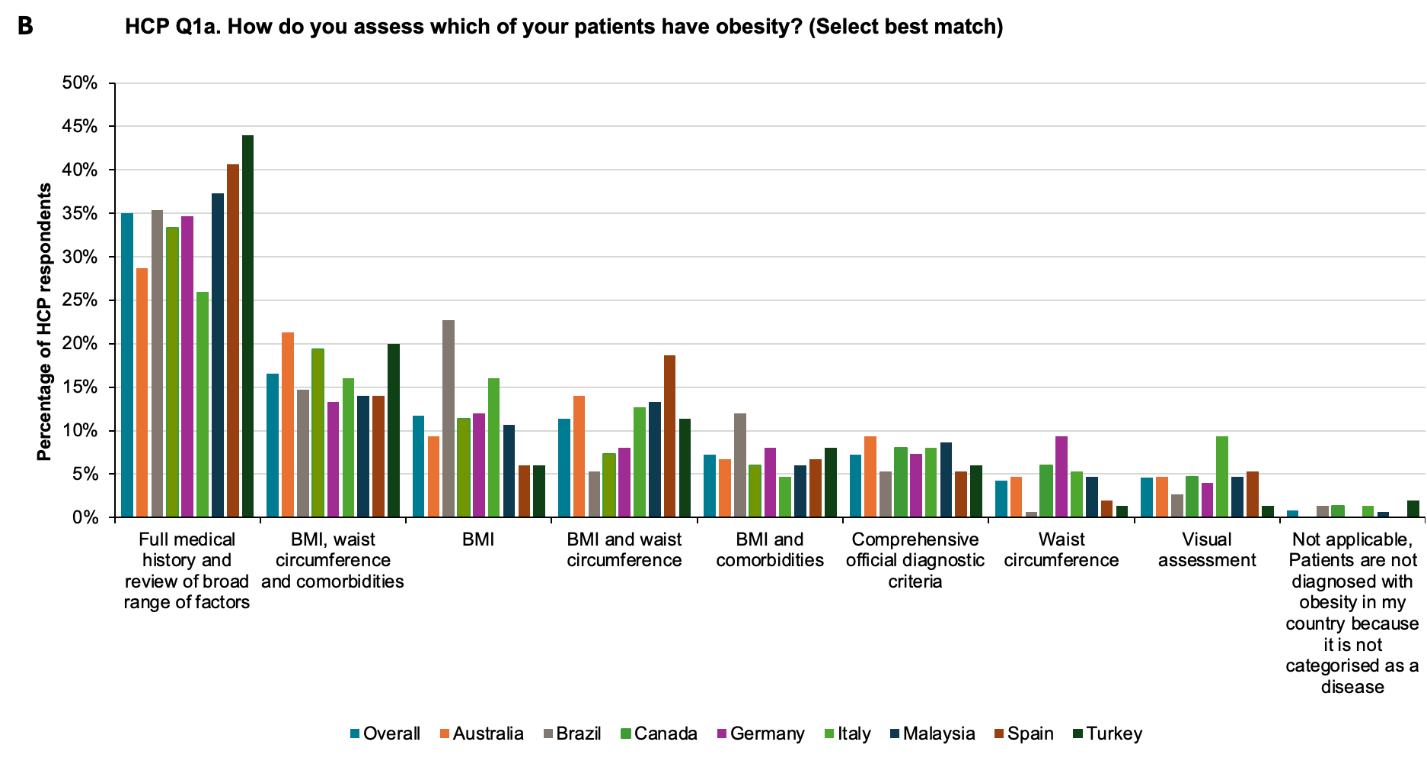


# **Table S2.** Mean percentages of HCPs reporting that patients’ diagnoses of obesity were or were not documented in their chart as a “chronic disease.”^a^

| **HCPs** | **Patients have been diagnosed and obesity^b^ is documented in their patient records as a chronic disease** | **Patients have been diagnosed with obesity^b^, but it is not documented as a chronic disease in their patient records** |
| --- | --- | --- |
| Overall (N = 1200) | 44.61% | 55.39% |
| Country |  |  |
| Australia (n = 150) | 46.12% | 53.88% |
| Brazil (n = 150) | 45.26% | 54.74% |
| Canada (n = 150) | 42.74% | 57.26% |
| Germany (n = 150) | 46.65% | 53.35% |
| Italy (n = 150) | 46.99% | 53.01% |
| Malaysia (n = 150) | 43.25% | 56.75% |
| Spain (n = 150) | 53.97% | 46.03% |
| Turkey (n = 150) | 39.84% | 60.16% |
| Specialty |  |  |
| GP/PCP (n = 323) | 44.21% | 55.79% |
| Practice nurse (n=323) | 44.84% | 55.16% |
| Endocrinologist (n = 265) | 45.07% | 54.93% |
| Cardiologist (n = 289) | 44.38% | 55.62% |

BMI, body mass index; GP, general practitioner; HCP, healthcare practitioners; PCP, primary care provider.

^a^ Based on “Q4. What percentage of your patients would you estimate have received an official diagnosis of obesity and have it documented in their patient records?” Answer was encoded as a sliding scale so that the combination of the two percentages had to add up to 100%.

^b^ Refers to patients with a formal obesity diagnosis and also applies to patients for whom weight alone/BMI has been documented in their records as part of routine checks.

# **Figure S5.** Mean percentages of patients with whom healthcare practitioners (HCPs) report feeling comfortable discussing obesity or actively engage in discussions about obesity.

Mean percentages were determined based on the proportions of respondents selecting each numerical answer option (i.e., 0%, 1–10%, etc.) and using the midpoint of each range as the value for options that were ranges. (A) By Specialty. (B) By country.


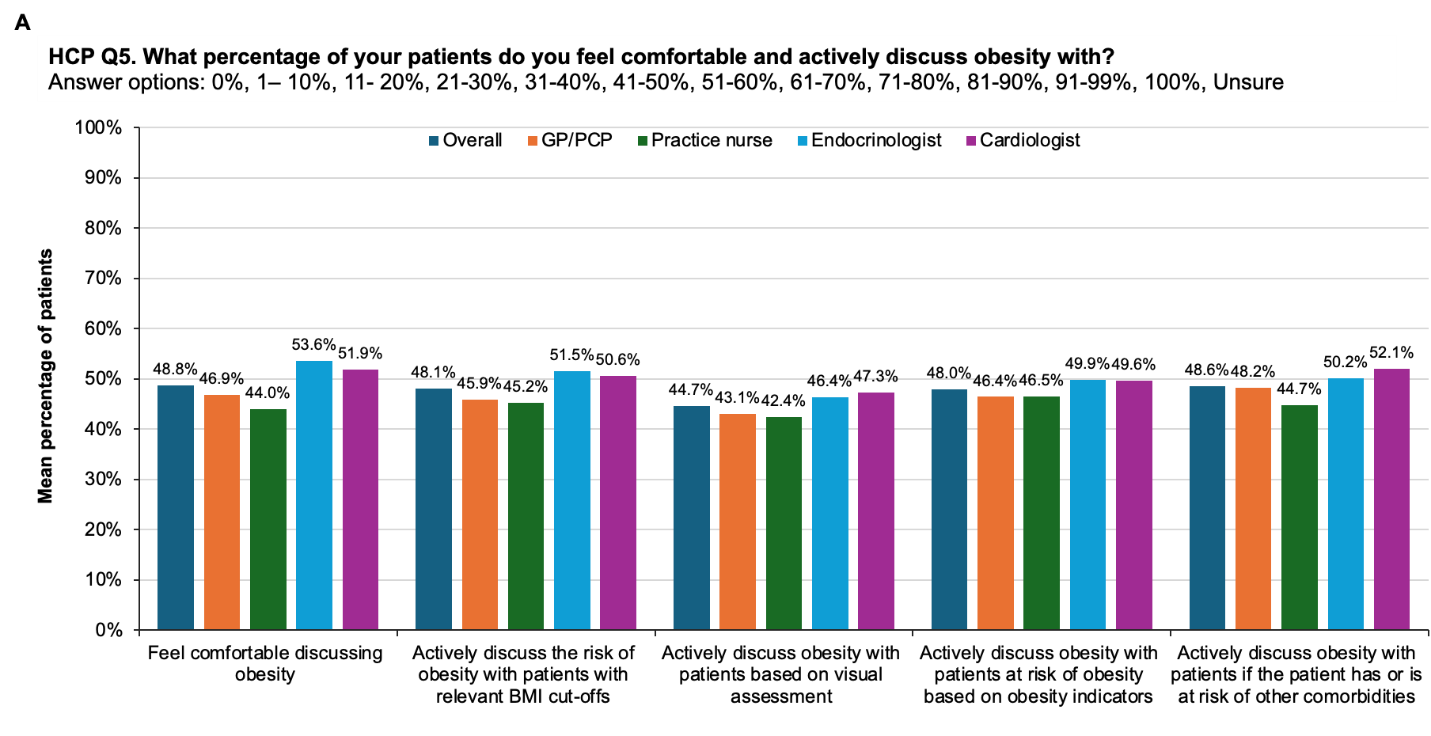


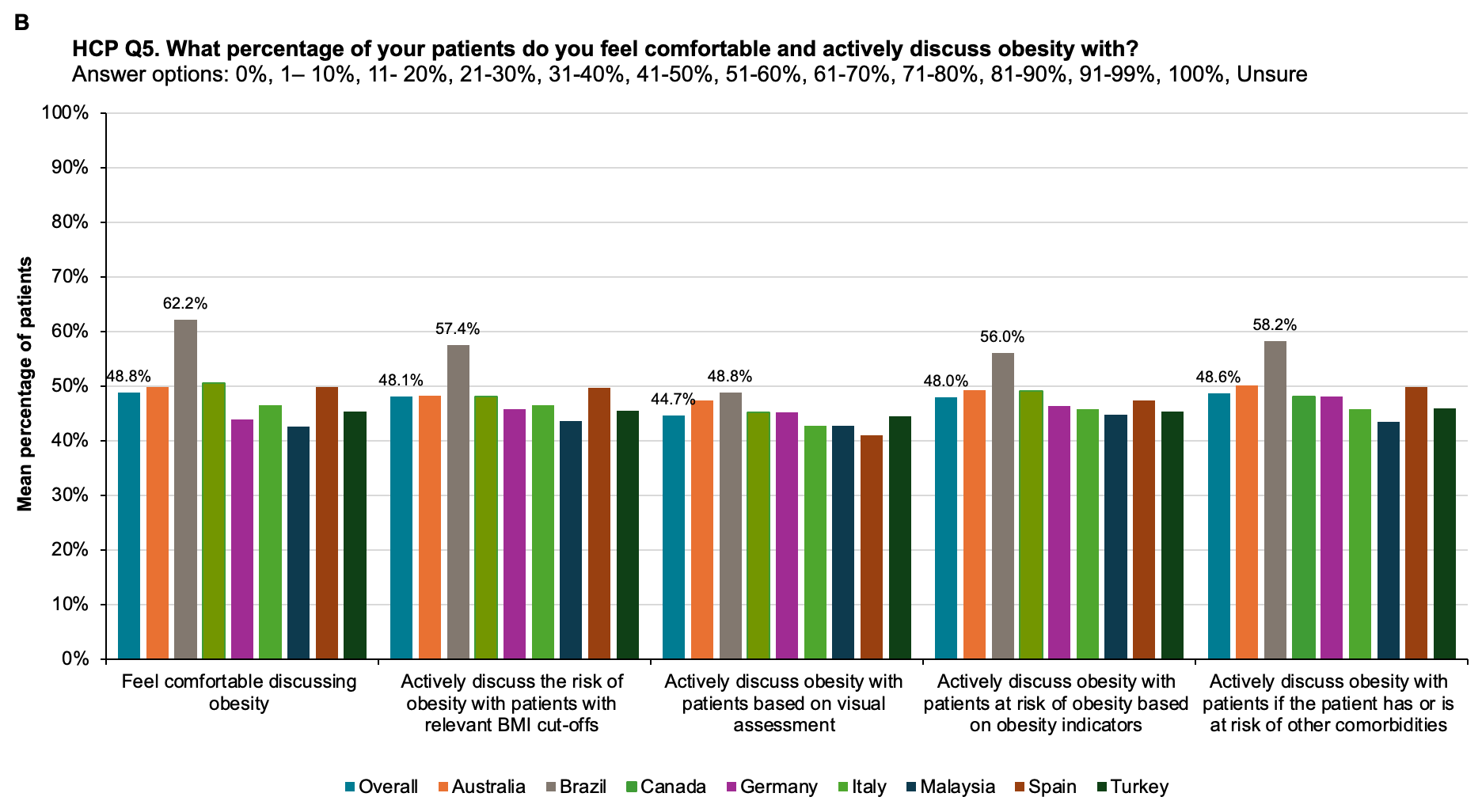


# **Figure S6.** Mean percentages of patients with obesity receiving referrals for the services listed as reported by healthcare practitioners (HCPs).

Mean percentages were determined based on the proportions of respondents selecting each numerical answer option (i.e., 0%, 1–10%, etc.) and using the midpoint of each range as the value for options that were ranges.


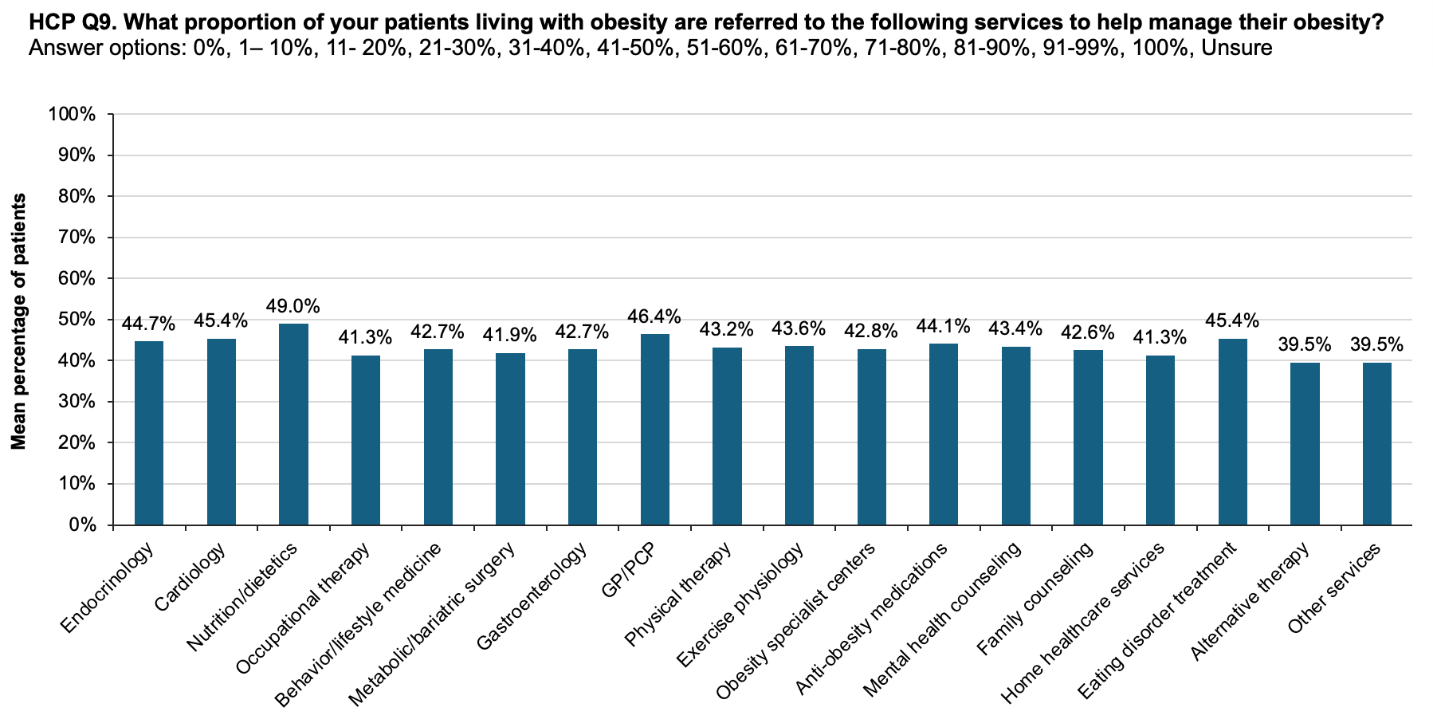


# **Figure S7.** Mean percentages of patients with obesity receiving the obesity interventions listed as reported by healthcare practitioners (HCPs).

Mean percentages were determined based on the proportions of respondents selecting each numerical answer option (i.e., 0%, 1–10%, etc.) and using the midpoint of each range as the value for options that were ranges. (A) By specialty. (B) By country.


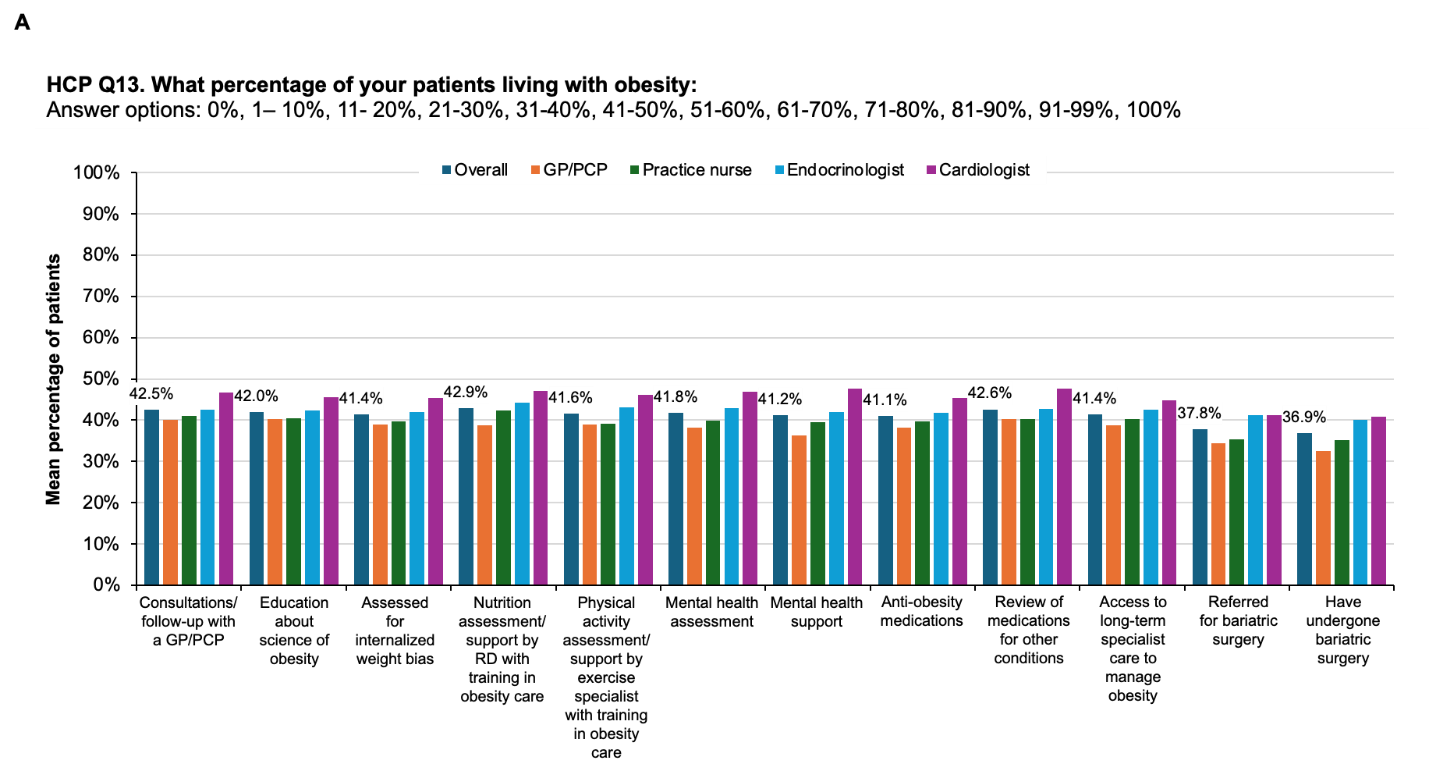

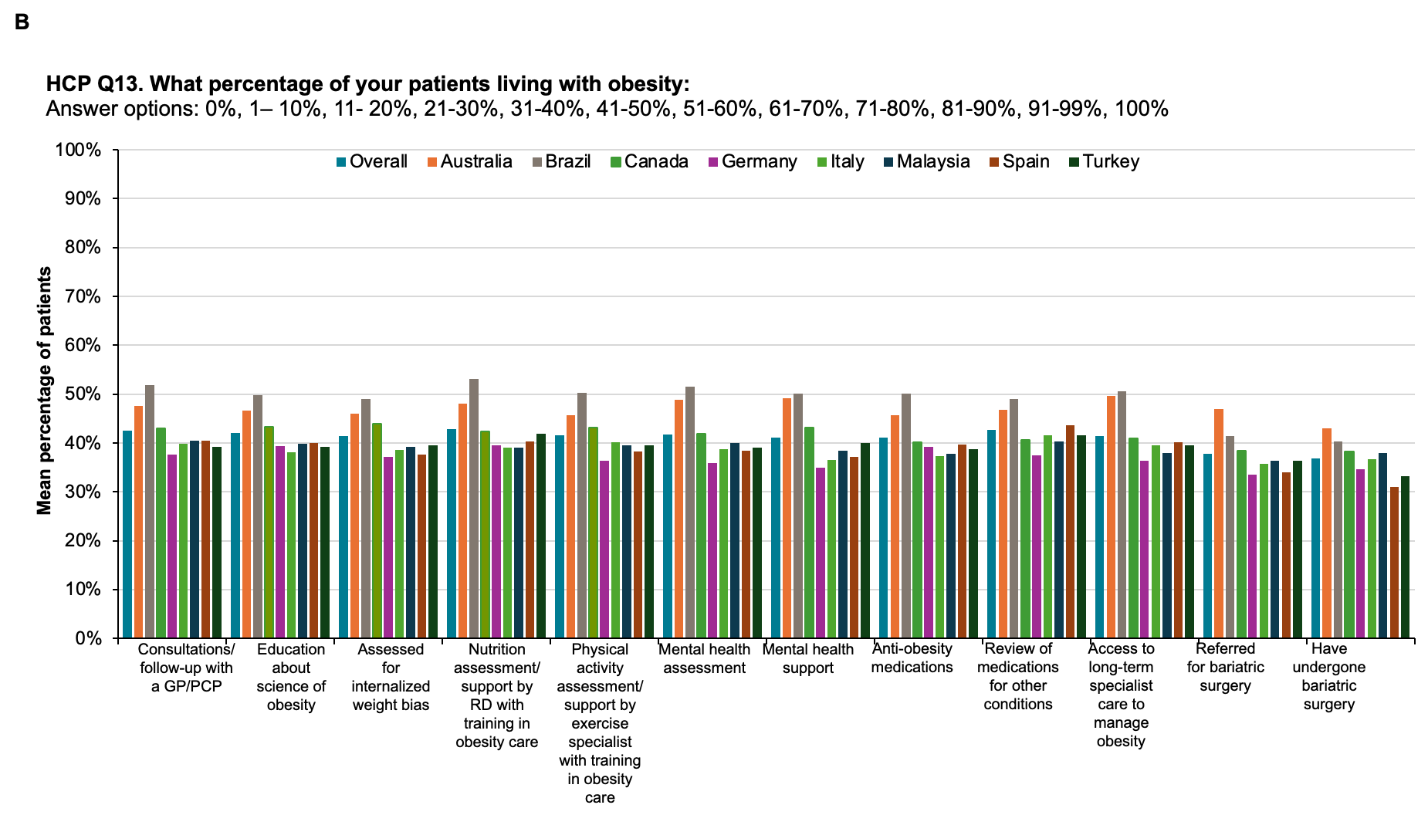

Supplement: Supplementary file 1 — Supporting Information S1 [file OSP4-11-e70033-s001.docx]
